# Loss of telomere silencing is accompanied by dysfunction of Polo kinase and centrosomes during *Drosophila* oogenesis and early development

**DOI:** 10.1371/journal.pone.0258156

**Published:** 2021-10-08

**Authors:** Valeriya Morgunova, Maria Kordyukova, Elena A. Mikhaleva, Ivan Butenko, Olga V. Pobeguts, Alla Kalmykova

**Affiliations:** 1 Institute of Molecular Genetics of National Research Centre “Kurchatov Institute”, Moscow, Russian Federation; 2 Federal Research and Clinical Center of Physical-Chemical Medicine, Moscow, Russian Federation; Virginia Polytechnic Institute and State University, UNITED STATES

## Abstract

Telomeres are nucleoprotein complexes that protect the ends of eukaryotic linear chromosomes from degradation and fusions. Telomere dysfunction leads to cell growth arrest, oncogenesis, and premature aging. Telomeric RNAs have been found in all studied species; however, their functions and biogenesis are not clearly understood. We studied the mechanisms of development disorders observed upon overexpression of telomeric repeats in *Drosophila*. In somatic cells, overexpression of telomeric retrotransposon *HeT-A* is cytotoxic and leads to the accumulation of *HeT-A* Gag near centrosomes. We found that RNA and RNA-binding protein Gag encoded by the telomeric retrotransposon *HeT-A* interact with Polo and Cdk1 mitotic kinases, which are conserved regulators of centrosome biogenesis and cell cycle. The depletion of proteins Spindle E, Ccr4 or Ars2 resulting in *HeT-A* overexpression in the germline was accompanied by mislocalization of Polo as well as its abnormal stabilization during oogenesis and severe deregulation of centrosome biogenesis leading to maternal-effect embryonic lethality. These data suggest a mechanistic link between telomeric *HeT-A* ribonucleoproteins and cell cycle regulators that ensures the cell response to telomere dysfunction.

## Introduction

The ends of linear chromosomes are protected from fusions and degradation by specialized nucleoprotein structures called telomeres. Telomeres promote genome stability by regulating DNA metabolism at chromosome ends. Telomere-dependent control of cellular proliferation ensures limited expansion of cells harboring chromosomal abnormalities [1–3]. This implies that there are mechanisms that link damaged telomeres to cell cycle checkpoints. The mechanisms of “telomeric signaling” appear to be based on the modulations of the levels of telomeric proteins that depend on the telomere state. Indeed, high levels of free telomeric proteins are a signal of shortened telomeres [4, 5]. Emerging evidence suggests that telomeric factors can control cellular targets elsewhere, thus acting as mediators of telomeric abnormalities [6]. For example, the release of Rap1 from shortened yeast telomeres and its ectopic binding to the non-telomeric targets results in transcriptional changes of non-telomeric genes [4]. Components of shelterin, the mammalian telomere protection protein complex, can also occupy non-telomeric sites and act as transcription factors [7, 8].

Telomeres can be transcribed into long telomeric repeat-containing RNAs (TERRA) in all taxa from yeast to mammals, suggesting a conserved role for telomeric RNAs [9]. Telomere transcription is highly dynamic, being regulated during the cell cycle and upon telomere dysfunction. Therefore, telomeric transcripts can be considered as possible candidates for telomeric signaling molecules. TERRA transcripts localize at telomeres in yeast and human cells [10, 11]. TERRA associates with the protein components of shelterin and telomeric chromatin, supporting its structural role in the recruitment of telomeric factors to telomeres [12]. Emerging evidence indicates that TERRA can localize outside of telomeres and regulate gene expression [13–15]. Because of the preferential nuclear localization of TERRA [16], investigation of TERRA partners has been limited to nuclear RNA and protein fractions. A large network of TERRA-interacting partners—including shelterin and chromatin proteins, telomerase components, transcription factors, DNA replication proteins, RNA-binding factors, and nuclear heterogenous ribonucleoproteins (RNPs)—has been discovered in the nuclei of mammalian cells [12, 15, 17–19].

TERRA has been found mainly in the nucleus, although cytoplasmic localization of shelterin proteins has been reported [20–22]. Interestingly, extranuclear TERRA foci have been detected in human cancer cell line after knockdown of retrotransposon Long Interspersed Nuclear Element 1 (LINE1) [23]. By studying *Drosophila* telomeres, we have found compelling evidence that *Drosophila* telomeric transcripts and their interacting proteins form RNP particles in the cytoplasm upon telomere dysfunction during oogenesis and early development [24], suggesting undiscovered functional implications of the cytoplasmic localization of telomeric factors.

The *Drosophila* genus and some other Diptera species have lost telomerase activity during evolution; their telomeres are maintained by transpositions of specialized telomeric retrotransposons (reviewed in [25–27]). Many functional and structural features of telomere homeostasis are conserved among species encoding telomerase or using telomeric retrotransposons. Such similarity could be explained by the general and pivotal role of telomeres in the maintenance of linear chromosomes and by the retrotransposon origin of telomerase reverse transcriptase [28, 29]. In both cases, telomeres are elongated by a reverse transcriptase. The protective complex terminin, a functional analogue of human shelterin, protects *Drosophila* chromosome ends from the DNA repair machinery and telomere fusions [30, 31]. Terminin consists of *Drosophila*-specific proteins binding telomeric DNA and single stranded overhang similar to the shelterin proteins. Studies of telomeric chromatin organization and regulation of telomeric repeat transcription in *Drosophila* and species possessing telomerase revealed many common mechanisms despite the structural differences [27, 32].

*Drosophila melanogaster* telomeres are elongated via the retrotransposition of three non-long terminal repeat (LTR) retrotransposons, *HeT-A*, *TART*, and *TAHRE*; non-autonomous *HeT-A* is the most abundant telomeric element [25]. Transcription of *Drosophila* telomeric retrotransposons was reported a decade before the discovery of TERRA [33]. *HeT-A*-encoded RNA-binding Gag protein performs typical telomeric functions: It localizes at telomeres, mediates specific targeting of the telomeric transcripts to chromosome ends, and prevents telomere fusions [24, 34, 35]. In wild type flies, the transcription of telomeric repeats is shut down primarily through the assembly of telomeric heterochromatin [36, 37]. In the *Drosophila* germline, the deposition and maintenance of repressive histone 3 mark, H3K9me3, and heterochromatin protein 1 (HP1) at telomeres is largely mediated by small Piwi-interacting RNAs (piRNAs). Accordingly, depletions of genes related to the heterochromatin assembly and piRNA pathway lead to the overexpression of telomeric elements and excessive telomere elongation [37–40]. We have also identified other factors related to different functional groups that downregulate the levels of *HeT-A* RNAs in the germline [41]. Among them the conserved RNA-binding arsenite-resistance protein 2 (Ars2) which acts also as a negative regulator of TERRA in human cells [17]. The deadenylase Ccr4–Not complex acts co-transcriptionally and mediates the degradation of *Drosophila* telomeric transcripts in telomere-associated bodies [42], whereas in human cells, deadenylases Ccr4 and Caf1 regulate telomerase RNA biogenesis in nuclear Cajal bodies [43]. These examples emphasize the similarity of regulatory processes despite the structural differences between the two types of telomeres.

It is believed that a balance between silencing mechanisms of *Drosophila* telomeric retroelements and the maintenance of appropriate levels of telomeric RNA template provides a proper telomere elongation and normal development [44]. The disruption of telomeric silencing in the germline leads to the accumulation of telomeric retrotransposon *HeT-A* RNPs in the cytoplasm of the germ cells and early embryos [24]. RNA-binding protein Egalitarian (Egl) [45, 46] was the first identified protein interacting with the cytoplasmic *HeT-A* RNP aggregates accumulated in the ovaries of the piRNA pathway mutants [24]. Egl mediates the proper localization of maternal mRNAs, and its ectopic localization within *HeT-A* Gag granules likely contributes to abnormalities of the embryonic axis specification, the phenomenon typical for the piRNA pathway mutants [47–49].

Maintaining telomere homeostasis during oogenesis is essential for genome stability during gametogenesis and early development, and telomere dysfunction is considered a potential biomarker for infertility in humans [50]. Depletion of any of the factors downregulating *Drosophila HeT-A* expression results in a highly similar phenotype characterized by mitotic defects during early development and embryonic lethality. This phenotype is accompanied by the accumulation of *HeT-A* RNPs consisting of *HeT-A* RNA and *HeT-A* Gag, an RNA binding protein, near centrosomes, which play the major role in mitotic spindle assembly in syncytial preblastoderm embryos [24, 41]. These observations inspired us to hypothesize about an unexpected role of cytoplasmic *HeT-A* RNPs in the embryonic lethality observed upon telomere dysfunction. Here, we have demonstrated the cytotoxicity of telomeric repeat *HeT-A* hyperexpression and revealed essential mitotic factors associated with cytoplasmic *HeT-A* RNA and Gag. Moreover, the depletion of Spindle E (SpnE), Ccr4 or Ars2 leads to accumulation of abundant *HeT-A* RNPs in the germline and is accompanied by impaired dynamics of Polo kinase, centrosome defects and aborted embryonic development. Our data suggest a mechanistic link between *HeT-A* mRNA, *HeT-A* Gag and cell cycle components, which could explain the severe developmental consequences of telomere dysfunction.

## Results

### Overexpression of telomeric retrotransposon *HeT-A* is cytotoxic and leads to the accumulation of *HeT-A* Gag near centrosomes

In our previous studies, we made a striking observation that the depletion of unrelated non-telomeric factors leading to *HeT-A* overexpression in the *Drosophila* ovaries, exhibited phenocopies during early development characterized by chromosome fusions, centrosome amplification and accumulation of *HeT-A* RNPs nearby centrosomes [24, 41]. However, this phenotype could be explained by the misregulation of various cellular mechanisms controlled by the studied factors rather than by telomere dysfunction. Therefore, we tested the cytotoxic effect of *HeT-A* overexpression in a normal cell line and *in vivo* in the wild type genetic background. For these experiments, we used pUAST-HeT-A-HA construct expressing *HeT-A* Gag protein tagged with HA and FLAG epitopes [24, 51].

**Fig 1:**
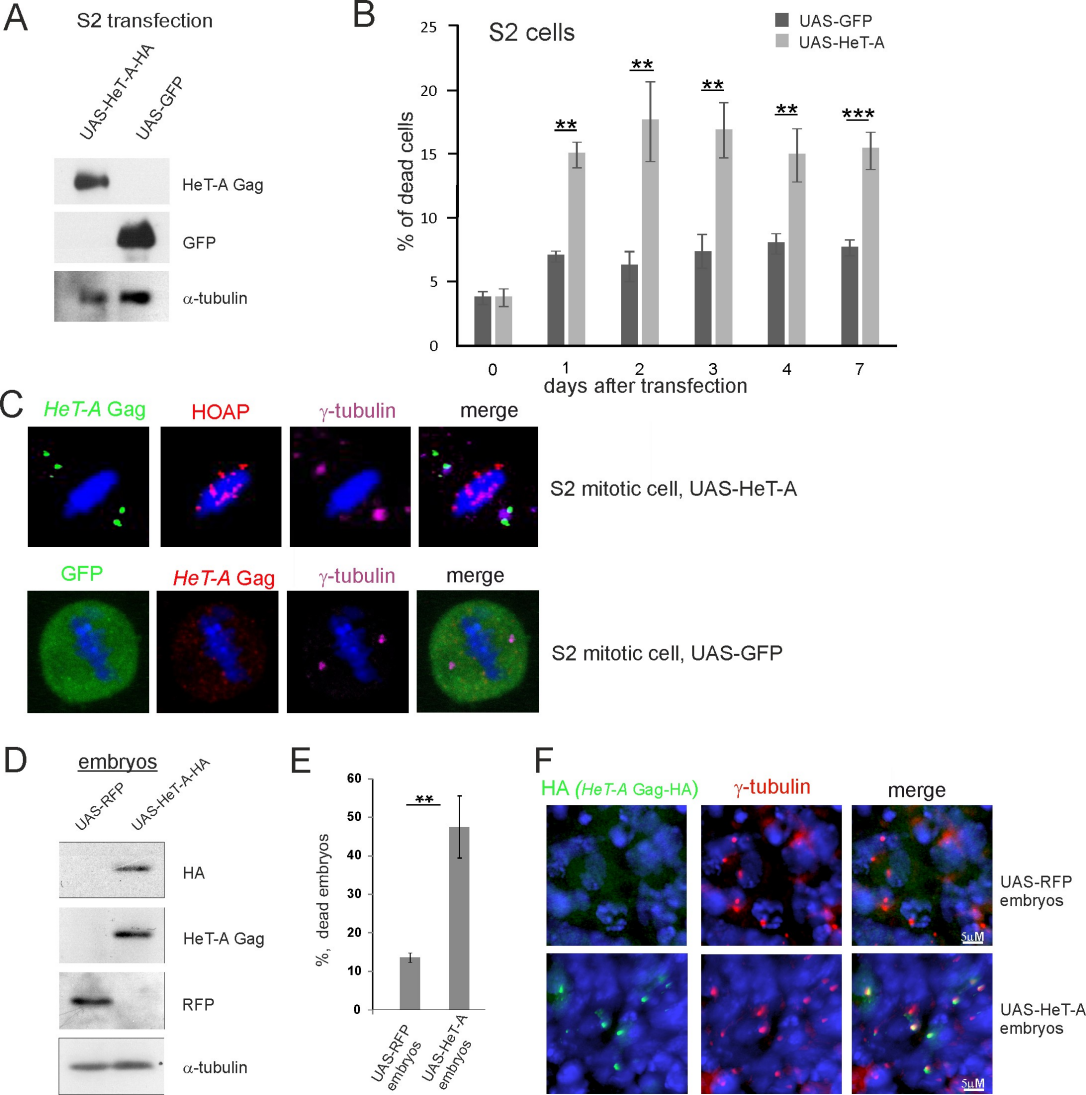
Induced overexpression of *HeT-A* in somatic cells is cytotoxic. (A) The samples of transfected S2 cells were separated by sodium dodecyl-sulfate polyacrylamide gel electrophoresis (SDS-PAGE) and analyzed by western blotting using the antibodies indicated on the right. The constructs used for transfection are indicated above the lanes. Co-transfection with pAct-GAL4 driver was used in both cases. (B) The proportion of dead cells present in the S2 cell suspension after transfection by constructs expressing *HeT-A* Gag or GFP. (C) *HeT-A* Gag (green), HOAP (red), and γ-tubulin (magenta) immunostaining was performed on S2 cells transfected with the construct expressing full-length *HeT-A* (upper panel). GFP (green), *HeT-A* Gag (red), and γ-tubulin (magenta) immunostaining was performed on S2 cells transfected with the construct expressing GFP (lower panel). Mitotic cells at metaphase are shown. DNA is stained with DAPI (blue). (D) Western blot analysis of embryonic extracts from strains expressing UAS-RFP or UAS-HeT-A-HA under the ubiquitous *da*GAL4 driver. The antibodies used for western blotting are indicated on the right. (E) The percentage of dead embryos laid by transgenic females expressing *HeT-A* Gag or RFP. (B and E) The SEM for four independent experiments was calculated. Asterisks indicate statistically significant differences (* P < 0.05 to 0.01, ** P < 0.01 to 0.001, *** P < 0.001, unpaired t-test). (F) *HeT-A* Gag-HA (green) and γ-tubulin (red) immunostaining was performed on 3–5-h-old embryos expressing *HeT-A* Gag-HA or RFP. DNA is stained with DAPI (blue).

*Drosophila* embryonic Schneider 2 (S2) cultured cells were transiently co-transfected with the pUAST-HeT-A-HA and pAct-GAL4 driver constructs to induce *HeT-A* overexpression. A similar experiment with the pUAST-GFP construct was performed as a control. The increased levels of *HeT-A* Gag and green fluorescent protein (GFP) proteins were validated by western blotting (Fig 1A). Death curves were analyzed for week after transfection. Increased cell death was observed starting from the second day after transfection with the *HeT-A*- but not the GFP-expressing constructs (Fig 1B). Immunostaining revealed a specific pattern of *HeT-A* Gag localization near centrosomes stained for γ-tubulin, a pericentriolar matrix (PCM) component, in all studied mitotic S2 cells (n = 22) transfected with the *HeT-A* construct; however, *HeT-A* Gag was not found at telomeres stained for the telomere-specific HOAP protein (Fig 1C). GFP was evenly dispersed in the cells (Fig 1C).

Next, we determined how overexpression of transgenic *HeT-A* in *Drosophila* embryos affects development. Expression of transgenic pUAST-HeT-A-HA was induced by GAL4 driven by the ubiquitous *daughterless* (*da*) promoter. Overexpression of the transgenic UAS-RedStinger driven by *da*GAL4 served as a control. There were high levels of *HeT-A* Gag-HA and red fluorescent protein (RFP) in 2–5-h-old embryos (Fig 1D). We next measured embryonic lethality. The survival of embryos with overexpression of *HeT-A* Gag-HA was significantly reduced compared with control embryos (Fig 1E). Immunostaining of 3–5-h-old embryos revealed centrosomal localization of *HeT-A* Gag-HA, whereas *HeT-A* Gag-HA was not detected in the embryos with RFP overexpression (Fig 1F). Similarly, expression of transgenic pUAST-HeT-A-HA induced by GAL4 driven by the neuronal *elav* promoter induced the accumulation of *HeT-A* Gag-HA around centrosomes of dividing neuroblasts in *D*. *melanogaster* larvae (S1 Fig).

We conclude that transgenic *HeT-A* overexpression in cultured cells and *in vivo* in the normal genetic background is cytotoxic and leads to cell death and embryonic lethality; this is accompanied by accumulation of *HeT-A* Gag-HA around centrosomes. Based on these observations, we suggested that centrosomal localization of *HeT-A* Gag may have a functional role, and *HeT-A* Gag may interfere with mitotic apparatus causing the arrest of cell divisions.

### *HeT-A* Gag associates with mitotic regulators in early *Drosophila* embryos

Due to the activity of the piRNA pathway, expression of transgenic *HeT-A* is strongly repressed in the germline in contrast to the somatic tissues, making the experiment with transgenic *HeT-A* overexpression in the wild type germline impossible [24]. Therefore, to study the effects of *HeT-A* overexpression in the germline, we further used mutations or germline knockdowns (GLKD) of factors that negatively regulate *HeT-A* expression: SpnE, a component of the piRNA pathway, Ccr4 deadenylase, encoded by the gene *twin*, and RNA-binding protein Ars2 (S2 Fig). During its lifecycle in the germline, *HeT-A* RNA forms RNP complexes with *HeT-A* Gag [24]; therefore, we used *HeT-A* Gag immunostaining or *HeT-A* RNA fluorescence in situ hybridization (FISH) to trace the distribution of *HeT-A* RNP.

**Fig 2:**
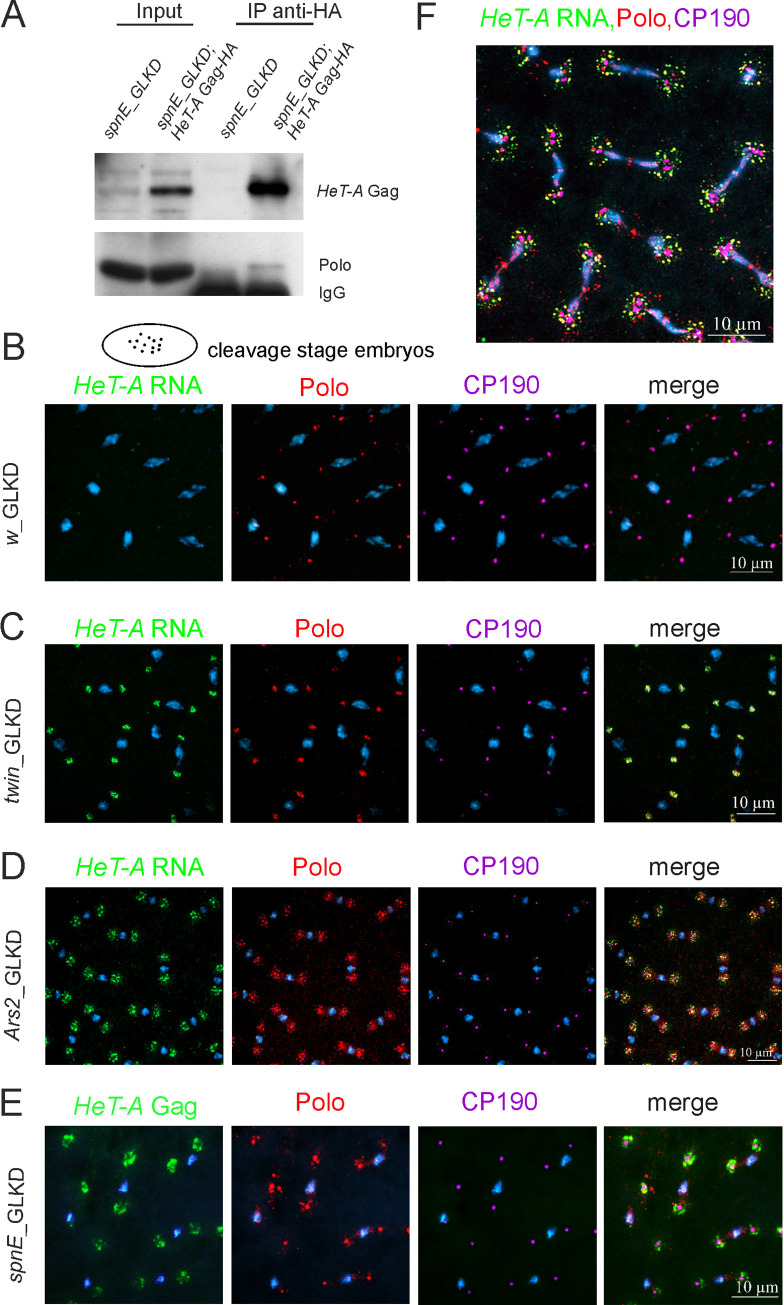
*HeT-A* ribonucleoproteins interact with Polo kinase in early *Drosophila* embryos. (A) Coimmunoprecipitation experiments were performed on extracts from 0–2-h-old *spnE*_GLKD embryos using an anti-HA antibody. The samples were separated by SDS-PAGE and analyzed by western blotting using the antibodies indicated on the right. The starting lysate (input) and immunoprecipitated (IP) probes are indicated; the antibodies used for co-IP are indicated above the IP lanes. (B-E) The images demonstrate the localization of *HeT-A* Gag or *HeT-A* RNA (green), Polo (phosphorylated form, red), and CP190 (magenta) in the control (B), *twin* (C), *Ars2* (D) and *spnE* (E) GLKD at metaphase in syncytial embryos. A cartoon of cleavage stage embryo is shown above the images. (F) The localization of *HeT-A* RNA (green), Polo (red), and CP190 (magenta) at anaphase in the *Ars2_*GLKD syncytial embryos. The blue color indicates DNA.

First, we studied the interaction partners of *HeT-A* RNPs in syncytial preblastoderm *Drosophila* embryos depleted of the piRNA pathway component *spnE*. In such embryos, the nuclei synchronously divide in a common cytoplasm, and abundant *HeT-A* RNPs surround the centrosomes [24, 41]. We performed affinity purification of the *HeT-A* Gag protein complex from the lysate of 0–2-h-old *spnE*_GLKD embryos expressing *HeT-A* Gag-HA using anti-hemagglutinin (HA) antibody–conjugated magnetic beads. In the control experiment, *spnE*_GLKD embryos did not carry the UAST-HeT-A-HA transgene. Purified proteins were separated by gel electrophoresis (S3 Fig) and analyzed by liquid chromatography coupled to mass spectrometry (LC-MS) (sample preparation and data analysis are described in the S2 File). The proteins involved in the regulation of cell division and centrosome maintenance were identified among potential partners of *HeT-A* Gag-HA (S1 Table). In this study, we have focused on Polo kinase, a conserved regulator of the cell cycle [52], which we have detected among *HeT-A* Gag-HA partners. We validated the association of Polo with *HeT-A* Gag-HA by co-immunoprecipitation (co-IP) performed using 0–2-h-old embryos of a transgenic strain with HA-tagged Gag *HeT-A* overexpression on the background of *spnE*_GLKD. The *HeT-A* Gag-HA protein complex was affinity purified using an anti-HA antibody and analyzed by western blotting. Polo was reproducibly co-purified with *HeT-A* Gag-HA (Fig 2A, S4A Fig), corroborating the MS data.

Polo is a highly dynamic enzyme, and the proper spatiotemporal control of its activity is crucial for correct cell divisions [53]. Polo colocalizes with centrosomal components throughout the entire embryonic mitotic cycle; in addition, Polo associates with the centromeres, mitotic spindle, and midbody [54]. Next, we asked whether the interaction of *HeT-A* Gag with Polo affects Polo localization during early embryogenesis. To this end, we performed Polo immunostaining in the syncytial embryos with *spnE*, *Ars2* and *twin* GLKD causing *HeT-A* overexpression and revealed substantial differences in Polo localization compared with wild type embryos.

As expected, Polo associated with centrosomes stained with centrosomal protein 190 (CP190) in the control embryos with the germline knockdown of the *white* gene (*w_*GLKD) encoding adult eye pigment (Fig 2B). In *spnE*, *Ars2*, and *twin* GLKD embryos, Polo was also detected at centrosomes, and there were also Polo aggregates randomly dispersed around the centrosomes and colocalized with endogenous *HeT-A* RNA and Gag-HA during syncytial cleavages (Fig 2C–2E). In anaphase of *Ars2*_GLKD, Polo/*HeT-A* RNA aggregates become more dispersed moving away from the centrosomes (Fig 2F). *HeT-A* RNA and Gag-HA also colocalized with Cdk1 in syncytial embryos with *spnE*, *Ars2*, and *twin* GLKD (S5 Fig). In the cortex of *spnE*_GLKD embryos, Cdk1 and *HeT-A* Gag-HA aggregates associate with free centrosomes (S5 Fig), the accumulation of which indicates mitotic defects in the syncytial embryo [55]. Western blotting did not reveal the changes in the Polo and Cdk1 protein levels in *spnE*_GLKD early embryos (S6 Fig).

In embryos overexpressing *HeT-A* we observe similar localization pattern between *HeT-A* RNA and *HeT-A* Gag and conclude that *HeT-A* RNPs interact with Polo and Cdk1, which probably leads to mislocalization of these mitotic kinases during early development.

### *HeT-A* RNA and Gag interact with mitotic kinases and centrosomal components during oogenesis

**Fig 3:**
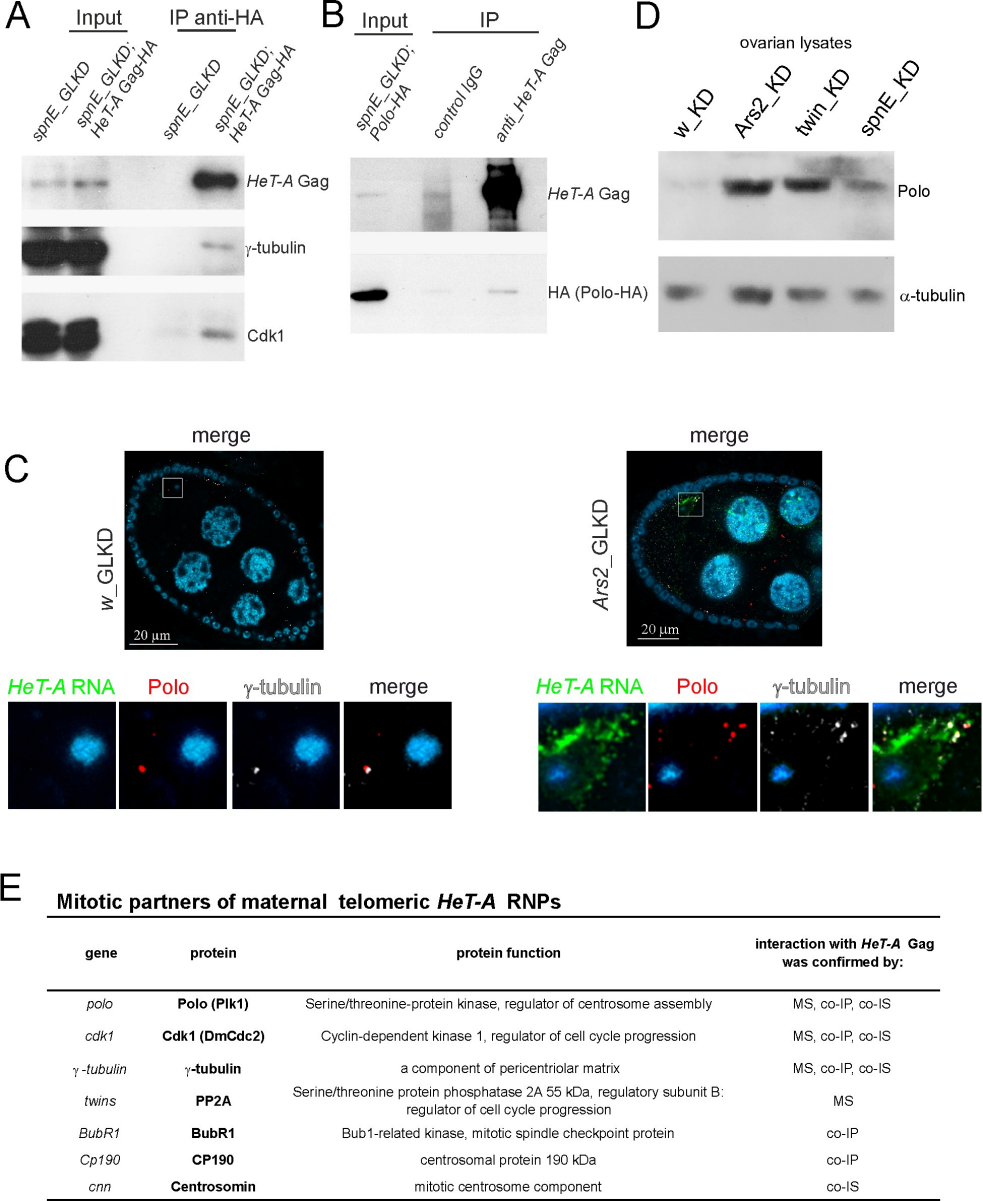
*HeT-A* ribonucleoprotein particles interact with Polo during oogenesis. (A) A coimmunoprecipitation experiment was performed on ovarian extracts from *spnE*_GLKD flies expressing *HeT-A* Gag-HA. (B) A co-IP experiment was performed on ovarian extracts from *spnE*_GLKD flies expressing Polo-HA. The samples were separated by SDS-PAGE and analyzed by western blotting using the antibodies indicated on the right. The starting lysate (input) and immunoprecipitated (IP) probes are indicated; the antibodies used for co-IP are indicated above the IP lanes. (C) *HeT-A* RNA FISH (green) combined with immunostaining of Polo (phosphorylated form, red) and γ-tubulin (grey) in the control (left panel) and *Ars2*_GLKD ovaries (right panel). Egg chambers at 9–10 stages of oogenesis are shown. The lower panels show the enlarged areas highlighted by squares. The blue color indicates DNA. (D) Western blot analysis of ovary lysates probed with anti-Polo antibody; α-tubulin was used as a loading control. (E) Mitotic partners of maternal *HeT-A* RNPs revealed by different methods. MS, mass spectrometry; co-IP, co-immunoprecipitation; co-IS, co-immunostaining.

Based on the observed interactions of *HeT-A* Gag-HA in syncytial embryos, we examined the mitotic partners of *HeT-A* Gag during oogenesis upon *HeT-A* overexpression. Co-IP was performed using ovary extracts of the transgenic strain expressing HA-tagged Gag *HeT-A* with *spnE_*GLKD. The *HeT-A* Gag-HA protein complex was affinity purified using an anti-HA antibody and analyzed by western blotting. Cdk1 kinase and γ-tubulin were co-purified with *HeT-A* Gag-HA (Fig 3A). The *HeT-A* Gag–Polo interaction in ovaries was confirmed by the co-IP experiment with anti-*HeT-A* Gag antibodies using *spnE_*GLKD flies expressing Polo-HA in the germline (Fig 3B). In addition, CP190 and the mitotic kinase BubR1 [56] were revealed in the ovarian *HeT-A* Gag-HA complex (S4B Fig).

Thus, abundant *HeT-A* RNPs interact with Polo kinase and other cell cycle regulators during oogenesis and early development (Fig 3E).

Polo is localized to the centrosomes throughout the mid-stages of *Drosophila* oogenesis, and its level sharply decreases at later stages of normal oogenesis [57]. At stages 9–10 of normal oogenesis, there was weak Polo staining barely detected in the vicinity of the oocyte nucleus (Fig 3C). In contrast to the normally reduced Polo levels at later stages, Polo was dispersed in the cytoplasm of the oocyte and co-localized with *HeT-A* RNA in *Ars2* and *twin* GLKD (Fig 3C, S7 Fig). The germline overexpression of UASg-Polo-HA transgene in *spnE*_GLKD resulted in the formation of Polo-HA aggregates co-localized with *HeT-A* RNA in the oocyte (S7 Fig). Accumulation of endogenous Polo in the ovaries of *spnE*, *twin*, and *Ars2* GLKD compared with control *w_*GLKD ovaries was validated by western blotting (Fig 3D). There was a more pronounced effect obtained by western blot analysis of 12–14 stage egg chambers of *spnE*_GLKD (S7 Fig).

Polo kinase activity is required for centrosome maturation and separation [58–60]. *polo* depletion causes a failure in the recruitment of centrosomin (CNN), γ-tubulin and CP190 to the centrosome [60, 61]. Given the crucial role for Polo in centriole biogenesis, we hypothesized that *HeT-A* RNP interactions with Polo and other regulatory cell cycle proteins lead to centrosome biogenesis defects during oogenesis.

### *HeT-A* overexpression is accompanied by centrosome dysfunction during oogenesis

The centrosomal structure is highly dynamic during oogenesis and early development. At the early stages of oogenesis, the centrosomes of the nurse cells move into the oocyte and form the microtubule-organization center (MTOC) near the nucleus [62].

**Fig 4:**
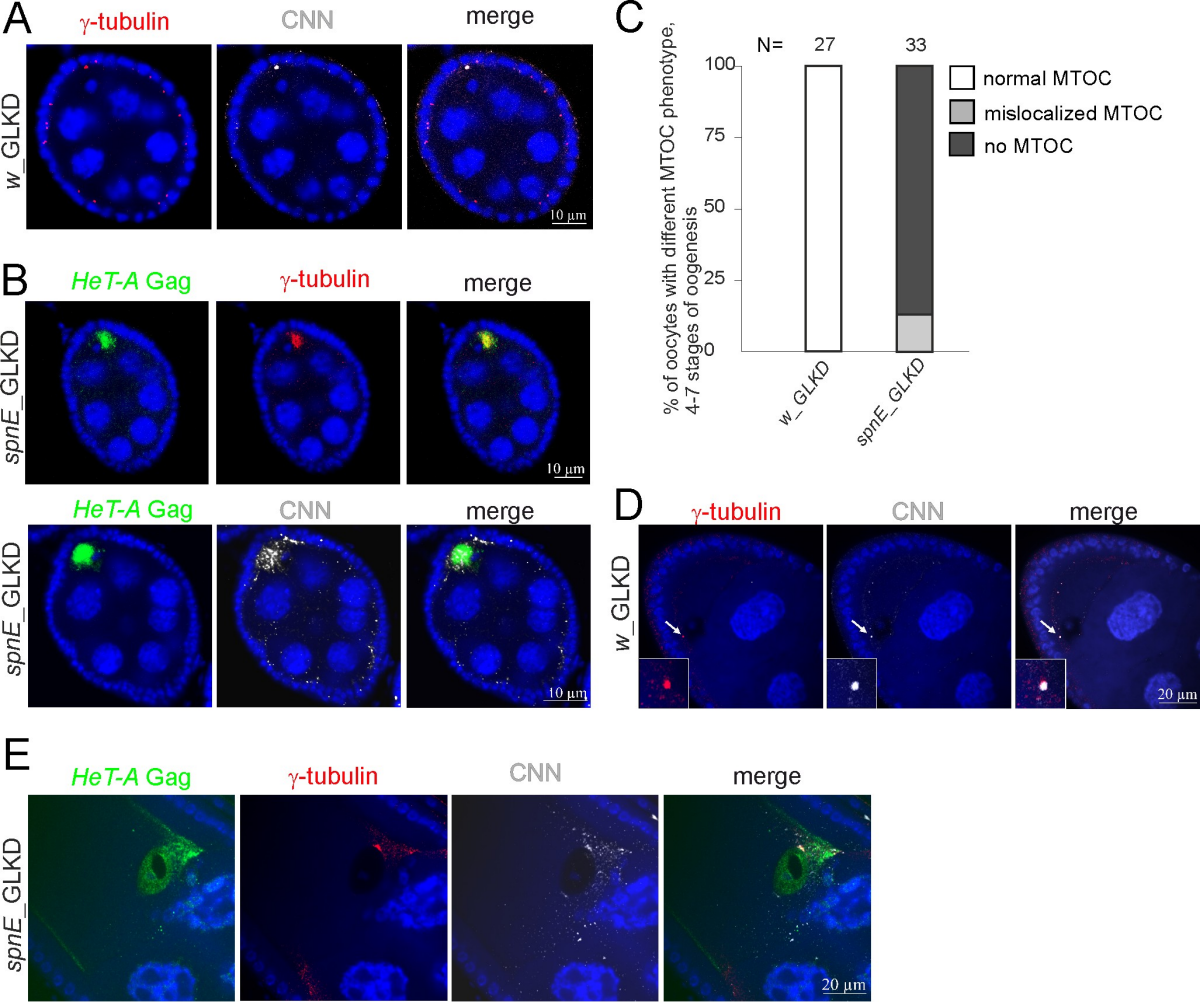
*HeT-A* overexpression caused by *spnE* knockdown is accompanied by centrosome dysfunction during oogenesis. (A) Co-immunostaining demonstrates colocalization of CNN (grey) and γ-tubulin (red) in the control ovaries (*w*_GLKD). (B) Co-immunostaining demonstrates the localization of *HeT-A* Gag (green), CNN (grey), or γ-tubulin (red) in *spnE*_GLKD ovaries. Stage 6 of oogenesis is shown (A, B). (C) Bar diagrams show the MTOC phenotype (%) observed at 4–6 stages of oogenesis in *spnE*_GLKD. (D) Co-immunostaining of CNN (grey) and γ-tubulin (red) shows the centrosome reduction at stage 9 of oogenesis in *w*_GLKD ovaries. (E) Immunostaining reveals the accumulation of *HeT-A* Gag (green) and mislocalized CNN (grey) and γ-tubulin (red) at stage 10 of oogenesis in *spnE*_GLKD. The blue color indicates DNA.

We studied the effect of *HeT-A* overexpression on MTOC formation during oogenesis. Immunostaining of CNN and γ-tubulin PCM was performed to monitor the state of the centrosome at different stages of oogenesis in the control and *spnE*_GLKD ovaries expressing *HeT-A* Gag-HA. In *w*_GLKD, the centrioles were clustered at a posterior pole of the oocyte, forming a strong focus of MTOC at stage 6 of oogenesis (Fig 4A). *spnE_*GLKD resulted in abnormal distributions of centrosomal proteins in the oocyte: CNN/γ-tubulin became dispersed in the oocyte at stage 6 of oogenesis and showed co-localization with abundant *HeT-A* Gag-HA (Fig 4B). Quantification of oocytes with a discrete MTOC revealed only a few of them in *spnE*_GLKD ovaries (Fig 4C). Even in these cases, the MTOC was unusually large and abnormally positioned (S8A Fig).

In many species, including *Drosophila*, centrioles disappear during oogenesis [63]. In *D*. *melanogaster*, the zygotic centriole is provided paternally, whereas maternal centrioles are eliminated during late stages of oogenesis. At these stages, Polo levels decrease sharply, resulting in a reduction and the eventual elimination of centrosomes [57, 64]. In *w*_GLKD, we observed a normal reduction of centrosomes at stage 9 of oogenesis (Fig 4D). In *spnE*_GLKD, centriolar protein staining persisted until stage 10 of oogenesis at the anterior pole in the vicinity of the nucleus and was partially co-localized with *HeT-A* Gag-HA (Fig 4E). Multiple enlarged and abnormally positioned MTOCs were frequently observed at stage 9 of oogenesis in *twin*_GLKD (S8B Fig). In *Ars2*_GLKD, centrosomes were abnormally amplified as shown by Polo–γ-tubulin immunostaining (Fig 3C).

Thus, we discovered an impaired centrosome biogenesis to be a common result of depletions of such functionally different factors as SpnE, Ccr4, or Ars2, all of which are characterized by the *HeT-A* overexpression phenotype. Moreover, *HeT-A* transcripts and Gag were associated with abnormally localized centrosomal proteins in the oocyte.

### *HeT-A* overexpression is accompanied by centriole dysfunction during late stages of oogenesis and early zygotic development

**Fig 5:**
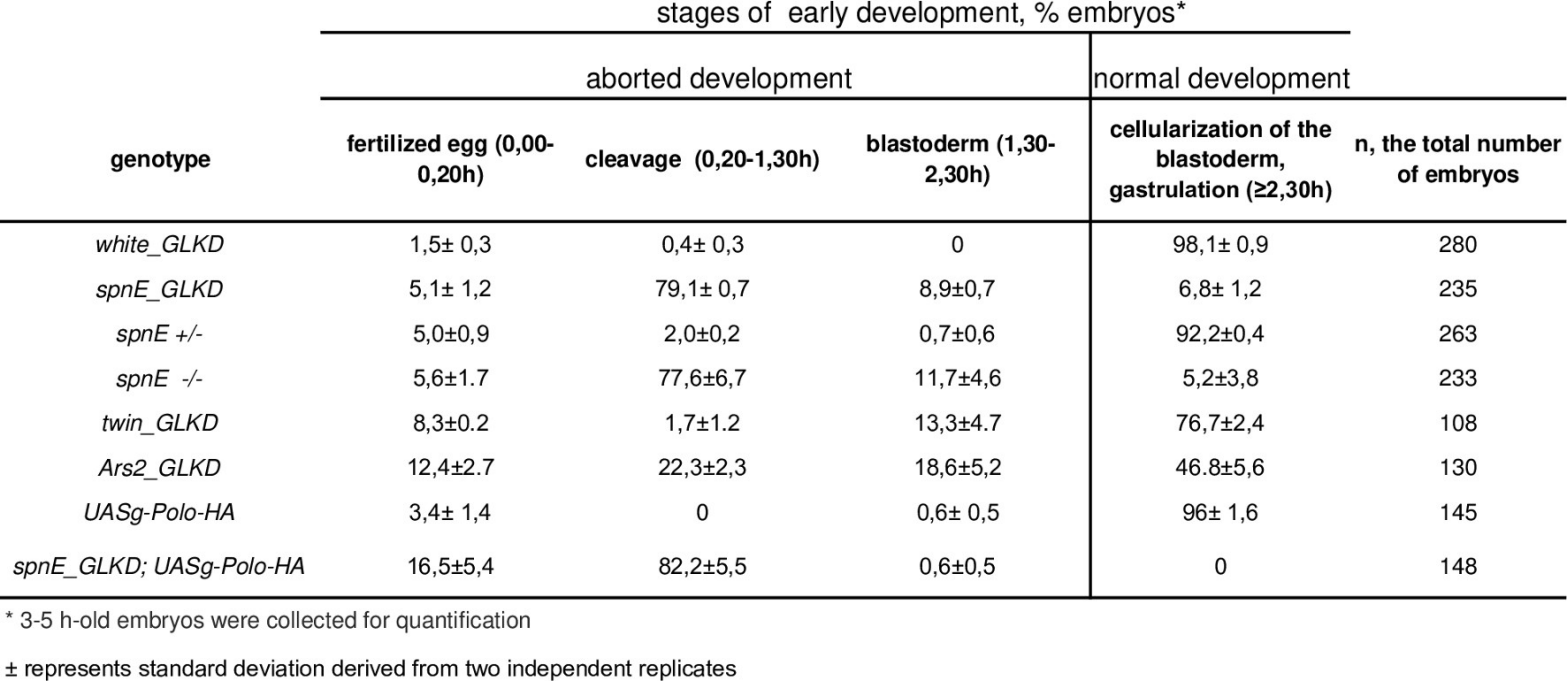
Maternal-effect embryonic lethality accompanying *HeT-A* overexpression.

Next, we addressed how depletion of three factors leading to *HeT-A* overexpression in the germline can affect early embryogenesis. We determined the stage of developmental arrest in *spnE*_GLKD, *spnE* mutants, *twin*_GLKD, and *Ars2*_GLKD embryos. To this end, we quantified the percentage of 3–5-h-old embryos arrested at different stages of embryonic development. We found that *spnE* GLKD or *spnE* mutations caused strong maternal-effect embryonic lethality (Fig 5). In those embryos that overcame the first zygotic divisions, there was developmental arrest at the early syncytial cleavage stage, resulting in > 80% early embryonic lethality. Of note, 10% and 35% of *twin*_GLKD and *Ars2*_GLKD embryos, respectively, were arrested before blastoderm formation.

It has been reported that the presence of maternal centrosomes caused by artificial Polo tethering to the centriole in the oocyte can lead to meiotic defects and developmental arrest during the initial zygotic divisions [57]. We observed that *HeT-A* overexpression was accompanied by abnormal accumulation of Polo until the late stages of oogenesis (Fig 3D). We therefore suggest that accumulation of Polo and centrosomal proteins during late stages of oogenesis could explain early developmental disorders in *spnE*, *twin* and *Ars2* GLKD flies characterized by *HeT-A* overexpression.

**Fig 6:**
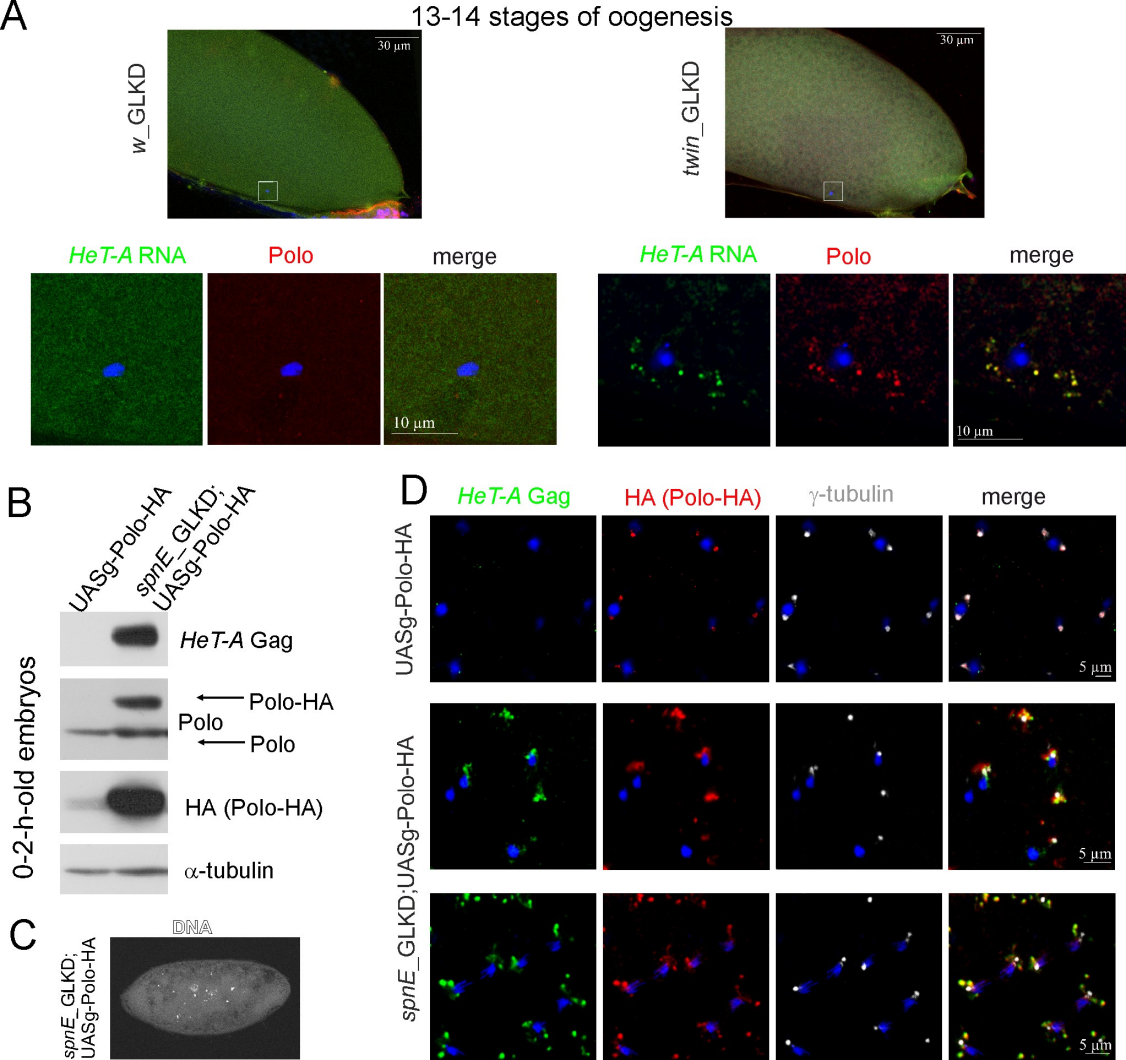
Retention of maternal Polo is mediated by its interaction with abundant telomeric *HeT-A* ribonucleoprotein particles. **(A)** *HeT-A* RNA FISH (green) combined with Polo (phosphorylated form, red) immunostaining in the control (*w*_GLKD) and *twin*_GLKD egg chambers at 13–14 stages. The upper images demonstrate the general view; the lower panels show the enlarged area around the oocyte nucleus obtained with a 63× objective lens. (B) Western blot analysis of 0–2-h-old embryo lysates shows the overexpression and accumulation of Polo-HA in *spnE*_GLKD embryos. The antibodies are indicated on the right. (C) DAPI staining of *spnE*_GLKD;UASg-Polo-HA embryos reveals developmental arrest at early syncytial cleavage stage. (D) Co-localization of *HeT-A* Gag (green) and Polo-HA (red) in the *spnE*_GLKD;UASg-Polo-HA syncytial embryos. γ-tubulin appears in grey, and DNA appears in blue.

We performed *HeT-A* RNA FISH combined with Polo and CNN immunostaining at stages 13–14 of oogenesis, which are normally characterized by centriole elimination. In contrast to the control oocytes (*w*_GLKD), which did not contain centrioles, we found Polo and CNN foci to be co-localized with *HeT-A* RNA around the oocyte nucleus in *twin*_GLKD (Fig 6A, S9 Fig).

We then asked whether overexpressing Polo kinase in the germline can affect the early embryonic lethality in the progeny of *spnE*_GLKD females. Using an embryonic developmental arrest test, we observed that the germline expression of UASg-Polo-HA enhanced the maternal-effect embryonic lethality of *spnE*_GLKD embryos (Fig 5). Embryonic lethality in *spnE*_GLKD;UASg-Polo-HA was predominantly observed during the initial cleavage divisions (Fig 6C). Western blotting demonstrated endogenous *HeT-A* Gag overexpression and strong Polo-HA accumulation in the 0–2-h-old embryos in *spnE*_GLKD;UASg-Polo-HA but not in UASg-Polo-HA embryos (Fig 6B). Immunostaining revealed abundant Polo-HA aggregates associated with endogenous *HeT-A* Gag dispersed around the centrosomes during the early embryonic cleavages (Fig 6D). We suggest that interaction of *HeT-A* Gag with abundant Polo-HA followed by ectopic retention of Polo-HA in the proximity of the centrosomes could be the main reason for centrosome dysfunction and the increased early developmental arrest rate observed in *spnE*-depleted embryos overexpressing Polo-HA.

Thus, the depletion of *spnE*, *twin* and *Ars2* characterized by *HeT-A* overexpression leads to Polo stabilization during oogenesis, a phenomenon that causes maternal centrosome dysfunction and severe mitotic defects during early development.

## Discussion

Using a *Drosophila* model, we have deepened our understanding of the mechanisms underlying the link between telomere dysfunction and defects in gametogenesis and early development. We have addressed a mechanism of cytotoxicity of abundant *HeT-A* RNPs accumulated in cells that overexpress telomeric repeats. Using different approaches, we have demonstrated that maternal telomeric RNPs encoded by the telomeric retrotransposon *HeT-A* interact with the key regulators of the cell cycle during oogenesis and early development (Fig 3E).

In the wild type ovarian germline, the expression of *HeT-A* is strictly downregulated by various factors, including the piRNA pathway [39, 40], HP1 chromodomain protein [37, 65], Woc and Trf2 transcription factors, Ars2 RNA-binding protein [41], and the Ccr4–Not deadenylase complex [42]. Recent findings have revealed the role of histone-modifying enzymes in the *HeT-A* repression in the germline [66, 67]. Depletions of each of these factors increase *HeT-A* RNA levels up to 1000 fold in the ovaries and cause severe ovary degeneration, patterning defects and aborted development [41, 47, 49, 68, 69]. Despite the fact that the factors listed above regulate numerous targets beyond *HeT-A*, we wondered whether *HeT-A* overexpression could be directly related to the observed phenotype. In this study, we have addressed the mechanism of developmental arrest upon *HeT-A* overexpression caused by the depletions of SpnE, a component of the piRNA pathway, Ars2, a key factor in RNA nuclear metabolism [70], and Ccr4 deadenylase, which plays an essential role in the control of maternal mRNA stability during oogenesis and early development [71]. Noteworthy, *HeT-A* is the only common transposon target of these three pathways. Indeed, SpnE, a component of the piRNA pathway, controls many transposon families [72], and Ccr4 deadenylase co-transcriptionally regulates *HeT-A* and actively transcribed transposons [42]. However, RNA-binding protein Ars2 downregulates the expression of telomeric but not other transposons [41, 73]. We cannot attribute mitotic defects solely to *HeT-A* overexpression and exclude the involvement of other common targets of SpnE, Ccr4 and Ars2. Nevertheless, our results strongly support an essential role of telomeres in impaired centrosome biogenesis in the ovaries of *spnE*, *twin* and *Ars2* GLKD. One may suggest that the observed high sensitivity of telomere signaling ensures a prompt cellular response to a wide range of genetic abnormalities.

Centrosomes are the key players in gametogenesis and the syncytial stage of embryogenesis. Their dynamics is regulated by mitotic kinases, and Polo plays a major role in centrosome maturation and separation [58]. We discovered the physical interactions between *HeT-A* Gag and the cell cycle kinases Polo and Cdk1 suggesting a mechanistic explanation for the role of *HeT-A* RNPs in the mitotic failure observed during early development after *HeT-A* overexpression [41]. Given the function of Polo in γ-tubulin and CNN recruitment to the centrosome, we suggested that the *HeT-A* Gag–Polo interaction may cause centrosome dysfunction. Supporting this idea, we observed severe defects in MTOC assembly during oogenesis: γ-tubulin and CNN were dispersed in the cytoplasm of oocytes instead of concentrating in the MTOC. However, because γ-tubulin, CNN, and CP190 also interact with *HeT-A* Gag and are found in Gag aggregates, the mechanism of centrosome dysfunction seems to be more complex. *HeT-A* Gag may directly or indirectly, through the assistance of Polo, interact with mitotic kinases and centrosome components, which collectively strongly affect centrosome dynamics. Abnormal accumulation of centrosomal proteins trapped in the *HeT-A* Gag complexes in the mature oocyte may explain the maternally determined embryonic lethality observed upon *HeT-A* overexpression. It seems that *HeT-A* RNP interactions with mitotic proteins do not play a role under normal conditions due to the extremely low levels of *HeT-A* expression [24], but they become a threat for development when *HeT-A* RNA levels exceed a certain threshold under telomere dysfunction conditions.

Analysis of maternal *HeT-A* Gag interactors identified a number of cell cycle proteins (Fig 3). Musaro et al. have reported a mechanistic link between *Drosophila* telomeres and components of anaphase checkpoint control [74]. In that work, studying the mechanism of metaphase arrest in the brain cells caused by unprotected *Drosophila* telomeres revealed that uncapped telomeres as well as the unattached kinetochores recruit the BubR1 kinase, which could inhibit anaphase onset. We found *HeT-A* Gag aggregates to be associated with Polo and Cdk1 in syncytial embryos around mitotic spindle poles, where components critical for proper mitotic progression are normally organized and orchestrated [75]. Frequently, *HeT-A* RNP and Polo/Cdk1 aggregates associate with free centrosomes in early embryos [41] (Fig 6D, S5E Fig). At the same time, detachment of γ-tubulin-positive centrosomes accompanied by elimination of the nuclei have been associated with compromised Polo function [76, 77]. These facts suggest the direct role of *HeT-A* RNP–Polo interactions in centrosome detachments during early development.

The mechanism of selective interaction of mitotic kinases with *HeT-A* Gag cytoplasmic granules is unclear. *HeT-A* Gag is phosphorylated, which is important for the protein stability [78]. It has been reported that Cdk1 activity produces the docking sites for Polo recruitment to its targets [79–81]. One may suggest that phosphorylation of *HeT-A* Gag by Cdk1 creates Polo docking sites leading to Polo targeting to the *HeT-A* Gag. Future studies will elucidate whether *HeT-A* Gag is a substrate of Polo and Cdk1 mitotic kinases.

What could be the evolutionary origin of the *HeT-A* Gag targeting to the centrosomes? Several viruses—including retroviruses and oncogenic viruses—have been shown to target the centrosome; moreover, viral proteins can directly interact with centrosomal proteins and alter their activity [82–86]. The evidence for cell cycle dysfunction promoted by viral proteins has been reported for the human immunodeficiency virus type 1 (HIV-1). HIV-1 Tat protein has been shown to induce apoptosis by interacting with the cell cycle proteins and suppressing Polo-like kinase 1 (Plk1) activity [87]. HIV-1 viral protein R (Vpr) localizes to the centrosome and modulates the activity of the ubiquitin ligase EDDDYRK2-DDB1^DCAF1^ or inhibits the Cdc25C phosphatase activity [88–90]. HIV-1 viral infectivity factor (Vif) causes dysfunction of Cdk1 and Cyclin B1 leading to cell cycle arrest [91]. Lastly, HIV-1 genomic RNA and Gag polyprotein colocalization near the centrosome is essential for viral particle assembly [92]. Centrosome targeting of retroviral GAG proteins has been described for mouse mammary tumor virus (MMTV), Mason-Pfizer monkey virus (M-PMV), and human foamy virus (HFV) [84, 93, 94]. It is believed that by targeting the centrosomal proteins, some viruses hijack their functions leading eventually to cell death or transformation. It is possible that *HeT-A*, as a retroelement, has inherited similar properties that have been adapted during evolution to the telomere signaling functions. In future studies, it will be important to determine the peptide sequences of *HeT-A* Gag responsible for the centrosomal targeting and interactions with the cell cycle and centrosomal proteins.

Interestingly, shelterin components being not of retrotransposon origin also localize at the centrosomes in human cells. Telomere repeat factor 1 (TRF1), a telomeric DNA-binding protein, localizes at mitotic spindles in human cancer cells [95] and mediates mitotic abnormalities via the Aurora-A kinase [96]. The telomeric factor tankyrase also localizes to both telomeres and centrosomes in HeLa cells [22]. Note that the association between telomeric proteins and the mitotic apparatus has been observed in cancer human cells for which telomere dysfunction is a typical hallmark. The molecular mechanism of the centrosome targeting of shelterin proteins is unclear. The retrotransposon origin of telomeres suggests that mechanisms of transposon control could be adopted for telomere regulation [26], and recent publications support this idea. Retrotransposon LINE1 knockdown leads to telomere dysfunction in human cancer cells [23, 97]. Moreover, LINE1 encoded proteins bind to telomeric ends and to TERRA [23]. One may suggest that the centrosome targeting of shelterin components could be mediated by retrotransposon proteins. It is tempting to speculate that interaction of the telomeric RNP with cell cycle regulators is an evolutionary conserved pathway present in both *Drosophila* and species possessing telomerase, and this process coupling telomere dysfunction and the cell cycle collapse can be considered as a telomeric checkpoint.

## Materials and methods

### *D*. *melanogaster* strains

The transgenic strain expressing *HeT-A* Gag protein tagged with HA and FLAG epitopes was described previously [24, 51]. As a control for the cell line experiments, the GFP open-reading frame (ORF) cloned into the pUASp-attB vector was used. Strain w[1118]; P{w[+mC] = UAS-RedStinger}6 (#8547, the Bloomington Drosophila Stock Center [BDSC]) was used as a control for the measurement of embryonic lethality. GLKD flies were F1 progeny of the genetic cross of a strain bearing a construct with short hairpin (sh)RNA (*spnE*_sh, #103913, the Vienna Drosophila Resource Center [VDRC]; *twin*_sh, #32490, BDSC; *Ars2*_sh, #106344, VDRC; *w*_sh, # 30033, VDRC, and a driver strain *P{UAS-Dcr-2*.*D}*. *v1*, *w*^*1118*^, *P{GAL4-nos*.*NGT}40*, #25751, BDSC). To induce tissue-specific expression of transgenic pUAST-HeT-A-HA, we used crosses with driver strains *P{GAL4-nos*.*NGT}40* (germline), *P{GAL4-da*.*G32}UH1* (ubiquitous) and *P{GawB}elav[C155]* (neuronal). The transgenic strain expressing poloORF-3xHA was from FlyORF (FO01229). The *spnE* mutant alleles were *spnE*^*1*^ and *spnE*^*hls3987*^.

### *Drosophila* cell culture experiments

*D*. *melanogaster* embryonic S2 cells were transfected using the FuGENE® HD Transfection Reagent (Promega) according to the manufacturer’s instructions. Co-transfection of pUAST-HeT-A-HA plasmid [51] or pUAST-GFP along with the driver plasmid pAct-GAL4 was performed to induce *HeT-A* or GFP expression. Twenty-four hours post-transfection, the cells were resuspended and seeded on coverslips for 1 h. Immunostaining was performed as described previously [98], except that the samples were incubated with secondary antibodies 1 h at room temperature. The number of viable cells in culture was determined by the trypan blue exclusion test [99] every 24 h for 7 days after transfection.

### Immunostaining and immunoprecipitation

Immunostaining and RNA FISH combined with immunostaining were carried out according to the previously described procedure [24]. A digoxigenin (DIG)-labeled antisense *HeT-A* riboprobe containing a fragment of the ORF (nucleotides 4330–4690 of GenBank sequence DMU06920) was used. Images were captured using a Zeiss LSM 900 confocal microscope and analyzed using ImageJ and Adobe Photoshop. Co-IP of *HeT-A* Gag-HA from embryo and ovary extracts was performed as described [24]. For co-IP of *HeT-A* Gag from the ovary extract, a Guinea pig anti-Gag antibody (kindly provided by Y. Rong) and Dynabeads Protein G magnetic beads (Invitrogen) were used (25 μL per sample).

### Antibodies

The following primary antibodies were used: rabbit and mouse anti-γ-tubulin (Sigma); rabbit anti-Cdk1/Cdc2 (Sigma); rabbit anti-CNN (kindly provided by T.L. Megraw; [100]); rat anti-CP190 (kindly provided by A. Golovnin; [101]); rabbit anti-Plk1 (phosphor-Thr210, LSBio, used for immunostaining and western blot analysis); mouse anti-Polo (monoclonal antibody MA81 used for western blot analysis was kindly provided by C. Sunkel; [102]); rabbit anti-BubR1 (kindly provided by C. Sunkel; [56]); rabbit anti-DsRed (Clontech); rabbit and mouse anti-HA (Cell Signaling Technology); mouse anti-GFP (Abcam); guinea pig anti-HOAP (polyclonal antibodies against full-length HOAP protein generated by PrimeBioMed, Skolkovo, Russia); guinea pig anti-Gag *HeT-A* (kindly provided by Y. Rong); rabbit anti-Gag *HeT-A* (kindly provided by E. Casacuberta; [103]); and mouse polyclonal anti-Gag *HeT-A* (generated in the Immunochemistry Laboratory, Branch of IBC RAS, Pushchino, Russia, using recombinant protein corresponding to the 1–375 amino acids of *HeT-A* Gag). Alexa Fluor–conjugated secondary antibodies with minimal cross-reactivity to IgG from non-target species (Jackson ImmunoResearch) were used (dilution 1:500).

### Quantification of embryonic phenotypes

Three hundred females were allowed to lay eggs on agar plates for 2 h. The flies were then removed, and the eggs were collected from the plates after 3 h. The 3–5-h-old embryos were fixed with methanol as described previously [104], incubated in phosphate-buffered saline (PBS) containing 0.5 μg/ml DAPI (4’,6-diamidino-2-phenylindole), and mounted in Vectashield Antifade Mounting Medium (Vector Laboratories). DNA staining of whole-mount embryos was used to visualize the stages of embryonic development according to [104].

## Supporting information

S1 Table Identifying proteins interacting with *HeT-A* Gag-HA in 0–2-h-old *Drosophila* embryos from mass spectrometry data. (XLSX)

S1 File Raw images. (RAR)

S2 File Supplementary methods. Mass spectrometry sample preparation and data analysis.
(PDF)

S1 Fig *HeT-A* Gag aggregates accumulate around centrosomes in mitotic neuroblasts of *Drosophila* larvae brain. *HeT-A* Gag-HA (red) and α-tubulin (green) immunostaining was performed on larvae brain of the *D*. *melanogaster* strains expressing RFP (upper panel, control) or *HeT-A* Gag-HA (lower panel). DNA is stained with DAPI (blue).
(TIF)

S2 Fig Characterization of strains used in the study. (A) Analysis of *HeT-A* RNA levels in the ovaries of indicated strains. RT-qPCR analysis of the *HeT-A* RNA levels normalized to RNA levels of *rp49* housekeeping gene. Bar diagrams show fold changes in steady-state *HeT-A* RNA levels in the ovaries of flies with the indicated genotypes relative to the control (*w_*GLKD). Error bars indicate SD (standard deviation) for three biological replicates. *spnE* mutants were *spnE*^*1*^*/spnE*^*hls3987*^. Heterozygous *spnE/+* flies were a mix of *spnE*^*1*^*/+* and *spnE*^*hls3987*^*/+*. (B) Efficiency of the germline knockdowns of SpnE, Ccr4 and Ars2. Western blot analysis of ovary extracts probed with indicated antibodies. α-tubulin staining was used as a loading control.
(TIF)

S3 Fig Proteins copurified with *HeT-A* Gag-HA in embryos. Coomassie brilliant blue staining of proteins copurified with *HeT-A* Gag-HA from 0-2-h-old *spnE*_GLKD embryos containing transgene expressing *HeT-A* Gag-HA. As a control, *spnE*_GLKD embryos without *HeT-A* transgene were used.
(TIF)

S4 Fig *HeT-A* Gag-HA interacts with cell cycle proteins. (A) Co-IP experiment performed on extracts from 0-2-h-old *spnE*_GLKD embryos expressing *HeT-A* Gag-HA reveals that Polo is co-purified with *HeT-A* Gag-HA. (B) Co-IP experiment performed on extracts from *spnE*_GLKD ovaries expressing *HeT-A* Gag-HA reveals that BubR1and CP190 are co-purified with *HeT-A* Gag-HA. Post-extraction insoluble pellet was loaded between Input and IP probes. The antibodies used for co-IP and western blotting are indicated on the right.
(TIF)

S5 Fig *HeT-A* RNA and Gag-HA interact with Cdk1 kinase in early *Drosophila* embryos. *HeT-A* RNA FISH (green) and coimmunostaining of Cdk1 (red) and CP190 (magenta) in the control (A), *Ars2*_GLKD (B) and *twin*_GLKD (C) early syncytial embryos. Lower panels in (C) show the enlarged area around the centrosome in *twin*_GLKD embryo. (D, E) Coimmunostaining of *HeT-A* Gag-HA (green), Cdk1 (red) and CP190 (magenta) in the *spnE*_GLKD is shown. Free centrosomes in the embryo cortex are shown (E). Blue, DNA.
(TIF)

S6 Fig Polo and Cdk1 protein levels in the early *Drosophila* embryos are not affected by *spnE*_GLKD. Western blotting of 0–2 h old embryo lysates shows the overexpression of *HeT-A* Gag and unchanged Polo and Cdk1 levels in *spnE*_GLKD embryos compared with the control (*w*_GLKD). Antibodies are indicated on the right. Anti-Plk1 antibodies (pThr210, LSBio) used here recognize phosphorylated form of Polo.
(TIF)

S7 Fig Polo dynamics is impaired in *spnE* and *twin* GLKD. (A) Colocalization of *HeT-A* RNA (green), Polo (phosphorylated form, red) and γ-tubulin (grey) in the oocyte of *twin*_GLKD at stage 9 is shown. Blue, DNA. (B) Accumulation of Polo-HA (red) and *HeT-A* Gag (green) in the oocyte of *spnE*_GLKD expressing Polo-HA at stage 6 of oogenesis is shown. Blue, DNA. (C) Western blotting confirms accumulation of phosphorylated Polo in *spnE_*GLKD egg chambers at 12–14 stages (rep1 and rep2, two biological replicates) compared with *w*_GLKD. The antibodies used are indicated on the right.
(TIF)

S8 Fig *HeT-A* overexpression is accompanied by centrosome dysregulation during oogenesis. (A) Coimmunostaining demonstrating colocalization of *HeT-A* Gag (green) and γ-tubulin (red) in *spnE*_GLKD ovaries at stage 5 of oogenesis is shown. (B) Immunostaining reveals accumulation of *HeT-A* Gag (green) and multiple γ-tubulin (red) and CNN (grey) foci at stage 9 of oogenesis in *twin*_GLKD. Blue, DNA.
(TIF)

S9 Fig Retention of maternal centrosomes is associated with telomere dysfunction in *twin*_GLKD. *HeT-A* RNA FISH (green) combined with CNN (gray) immunostaining in *twin*_GLKD at stage 13–14 egg chamber. The lower panel is a blow up of the area around the oocyte nucleus (the upper panel). DNA, blue.
(TIF)
