# Supplementary material for: Loss of telomere silencing is accompanied by dysfunction of Polo kinase and centrosomes during Drosophila oogenesis and early development
Source: PLoS One. 2021 Oct 8;16(10):e0258156. doi: 10.1371/journal.pone.0258156 (PMC8500440; doi:10.1371/journal.pone.0258156)
Supplement: S2 File — Mass spectrometry sample preparation and data analysis. (PDF) [file pone.0258156.s003.pdf]

Loss of telomere silencing is accompanied by dysfunction of Polo kinase and centrosomes during *Drosophila* oogenesis and early development

Valeriya Morgunova[1], Maria Kordyukova[1], Elena A. Mikhaleva[1], Ivan Butenko[2], Olga V. Pobeguts [2], and Alla Kalmykova[1]

[1] Institute of Molecular Genetics of National Research Centre “Kurchatov Institute”, Moscow 123182, Russian Federation
[2] Federal Research and Clinical Center of Physical-Chemical Medicine, Moscow, Russian Federation

Supplementary methods

Mass spectrometry sample preparation and data analysis

In-gel trypsin digestion. Co-immunoprecipitated proteins were fractionated by one-dimensional electrophoresis (12% SDS PAAG) under denaturing conditions. Gels were stained with the Coomassie blue. The strips of gel were cut in small pieces of approximately 1x1 mm and washed in 30 mM of sodium thiosulfate (Sigma) and 100 mM of potassium ferricyanide (Sigma) at 1: 1 ratio. Then, 10 mM DTT (BioRad) in 100 mM NH4HCO3 buffer was added to gel pieces for reduction of protein disulfide bonds. After 30 min incubation at 56° C, this solution was discarded and 55 mM iodoacetamide (IAA, BioRad) in 100 mM NH4HCO3 buffer was added for its alkylation. After 20 min incubation in darkness at room temperature, the solution was discarded and 10 mM DTT in 100 mM NH4HCO3 buffer was added to remove the remaining IAA. In 15 minutes, the solution was discarded and the gel pieces were completely dried with 100% acetonitrile (Sigma). For trypsinolysis, trypsin solution (Trypsin Gold, Mass Spectrometry Grade, Promega) (25 ng/µl in 40 mM NH4HCO3 and 10% acetonitrile) was added to the dried gel pieces and incubated for 30 min on ice, and then at least 16 hours at 37°C. The peptides were sequentially extracted with 0.1% trifluoroacetic acid (TFA, Sigma) (1:1), 0.1% TFA (1:1) and 50% acetonitrile (1:1). Then the samples were sonicated in an ultrasonic bath for 10 minutes and incubated for 60 minutes at room temperature. Peptide extracts were mixed,

dried in a SpeedVac (Labconco) and dissolved in 50 μl 0.1% TFA and 3% acetonitrile. Peptide extract was desalted using a Discovery DSC-18 Tube (Supelco) according to the manufacturer protocol. The peptide concentrations were determined using BCA Protein Assay (Sigma). After purification, the samples were dried and resuspended in 0.1% TFA and 3% acetonitrile to the final concentration of 5 μg/ml.

Liquid chromatography–mass spectrometry (LC-MS) analysis. Analysis was performed on a TripleTOF 5600+ mass-spectrometer with a NanoSpray III ion source (ABSciex, Canada) coupled to a NanoLC Ultra 2D+ nano-HPLC system (Eksigent). The HPLC system was configured in a trap-elute mode. Sample loading solvent and solvent A were 98.9% water with 1% methanol and 0.1% formic acid (v/v) and solvent B was 99.9% acetonitrile with 0.1% formic acid (v/v). Samples were loaded on a home-made trap column (100 μm ID x 1 cm, packed with Aeris Peptide XB-C18 sorbent, 2.6 μm particle size) at a flow rate of 5 μl/min over 5 min and eluted through home-made separation column (75 μm ID x 15 cm, the same sorbent as in trap column) at a flow rate of 300 μl/min. The gradient was from 10% to 40% of solvent B during 60 min. The column and the precolumn were regenerated between runs by washing with 90% of solvent B for 9 min and equilibrated with 10% of solvent B for 13 min.

Mass spectra were acquired in a positive ion mode. Information-dependent mass-spectrometer experiment included 1 survey MS1 scan followed by 50 dependent MS2 scans. MS1 acquisition parameters were as follows: mass range for analysis and subsequent ion selection for MS2 analysis was 300-1250 m/z, signal accumulation time was 250 ms. Ions for MS2 analysis were selected on the basis of intensity with the threshold of 100 cps and the charge state from 2 to 5. MS2 acquisition parameters were as follows: resolution of quadrupole was set to UNIT (0.7 Da), measurement mass range was 100-2000 m/z, optimization of ion beam focus was to obtain maximal sensitivity, signal accumulation time was 100 ms for each parent ion. Collision activated dissociation was performed with nitrogen gas with collision energy ramping from 25 to 55 V within 100 ms signal accumulation time. Analyzed parent ions were sent to

dynamic exclusion list for 15 sec after 2 MS2 events in order to get the next MS2 spectra of the same compound around its chromatographic peak apex (minimum peak width throughout the gradient was about 30 s).

LC-MS data analysis. For protein identification and semi-quantitative spectral counting all LC-MS/MS data was searched against Drosophila melanogaster reference proteome (UniProt,UP000000803, including isoforms) accompanied with common contaminant proteins. Identification was performed with ProteinPilot (version 4.5, Sciex) in identification mode with the following parameters: Cys alkylation by iodoacetamide, trypsin digestion, TripleTOF 5600 instrument, FDR analysis and thorough I.D. search with a detected protein threshold of 95.0%. Protein identification were considered significant if both estimated local false discovery rate was 1% or lower and at least two different peptides were identified for protein with Confidence score above 95%. Spectral counting was performed with in-house script under emPAI protocol with only tryptic peptides with local FDR of 1% or lower taken into account.
