# Supplementary figures and images for: Loss of telomere silencing is accompanied by dysfunction of Polo kinase and centrosomes during Drosophila oogenesis and early development
Source: PLoS One. 2021 Oct 8;16(10):e0258156. doi: 10.1371/journal.pone.0258156 (PMC8500440; doi:10.1371/journal.pone.0258156)

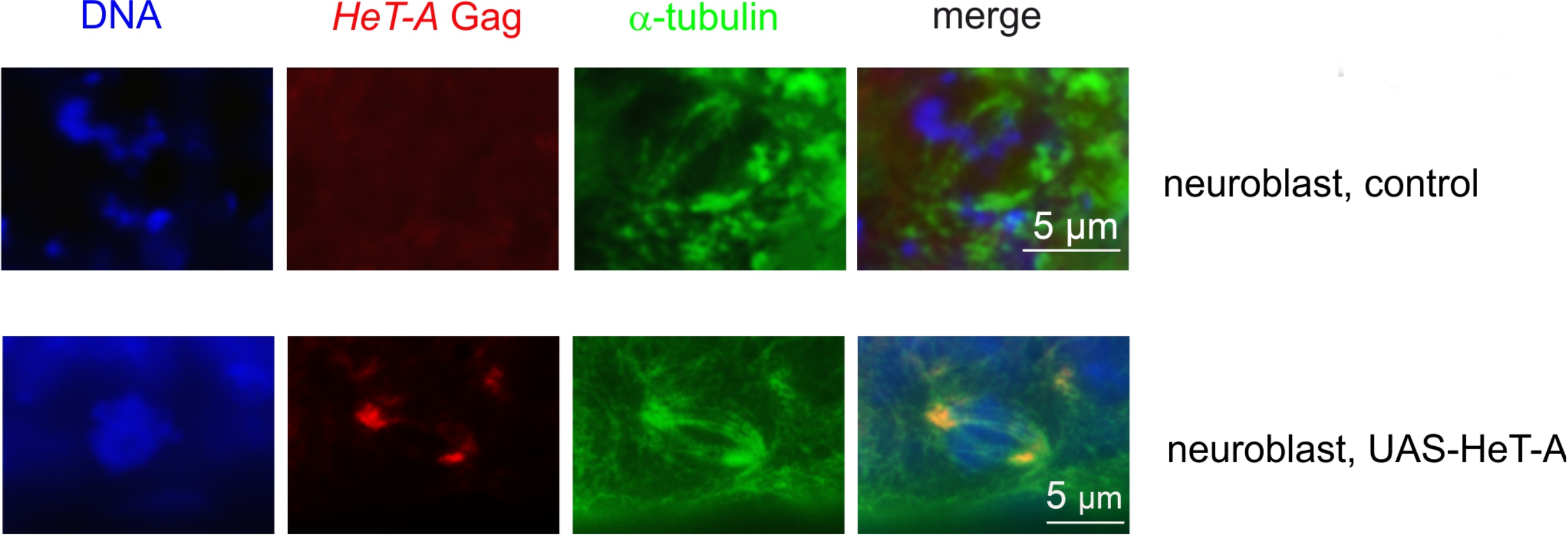

Supplement: S1 Fig — HeT-A Gag-HA (red) and α-tubulin (green) immunostaining was performed on larvae brain of the D. melanogaster strains expressing RFP (upper panel, control) or HeT-A Gag-HA (lower panel). DNA is stained with DAPI (blue). (TIF) [file pone.0258156.s004.tif]

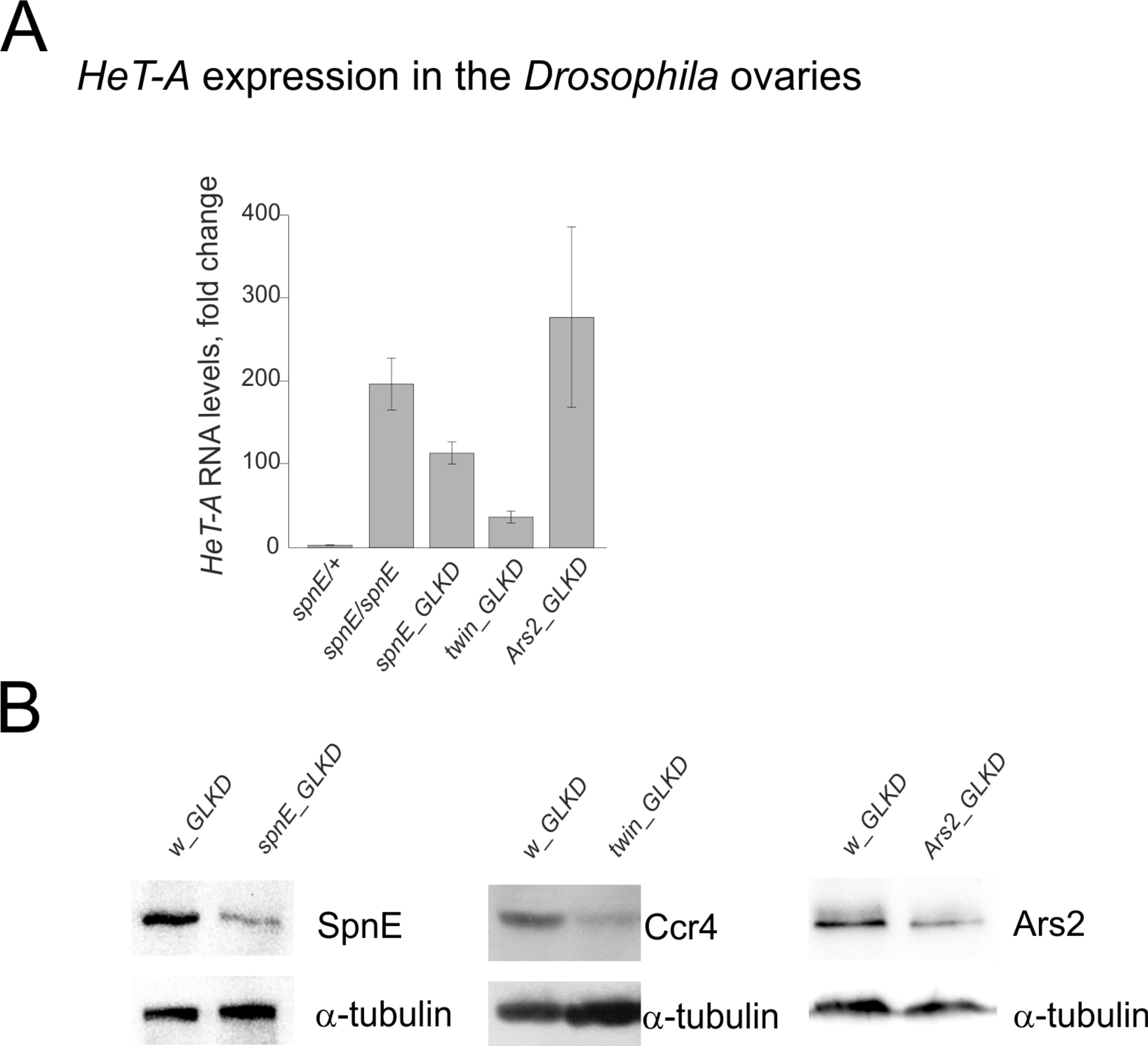

Supplement: S2 Fig — (A) Analysis of HeT-A RNA levels in the ovaries of indicated strains. RT-qPCR analysis of the HeT-A RNA levels normalized to RNA levels of rp49 housekeeping gene. Bar diagrams show fold changes in steady-state HeT-A RNA levels in the ovaries of flies with the indicated genotypes relative to the control (w_GLKD). Error bars indicate SD (standard deviation) for three biological replicates. spnE mutants were spnE1/spnEhls3987. Heterozygous spnE/+ flies were a mix of spnE1/+ and spnEhls3987/+. (B) Efficiency of the germline knockdowns of SpnE, Ccr4 and Ars2. Western blot analysis of ovary extracts probed with indicated antibodies. α-tubulin staining was used as a loading control. (TIF) [file pone.0258156.s005.tif]

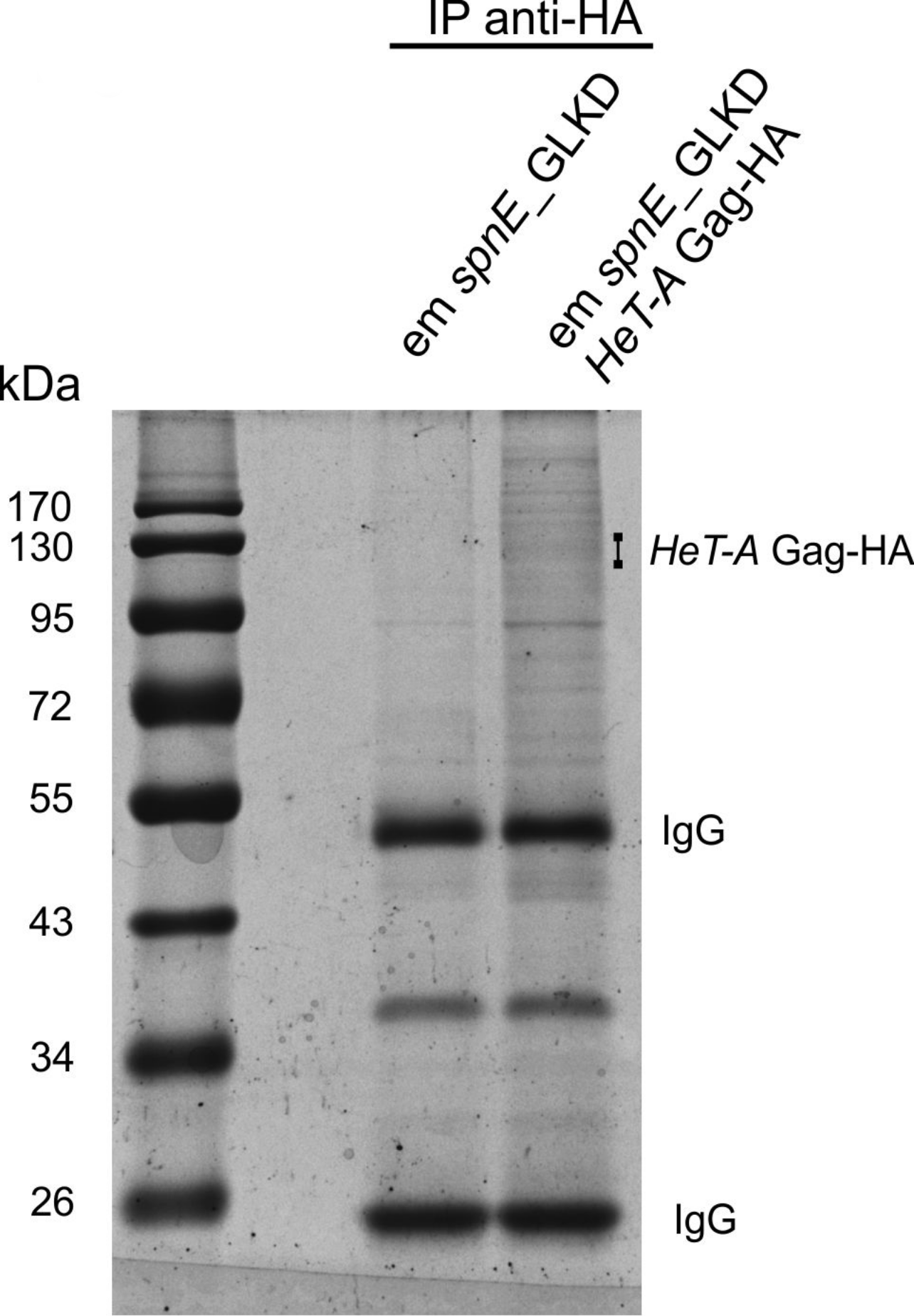

Supplement: S3 Fig — Coomassie brilliant blue staining of proteins copurified with HeT-A Gag-HA from 0-2-h-old spnE_GLKD embryos containing transgene expressing HeT-A Gag-HA. As a control, spnE_GLKD embryos without HeT-A transgene were used. (TIF) [file pone.0258156.s006.tif]

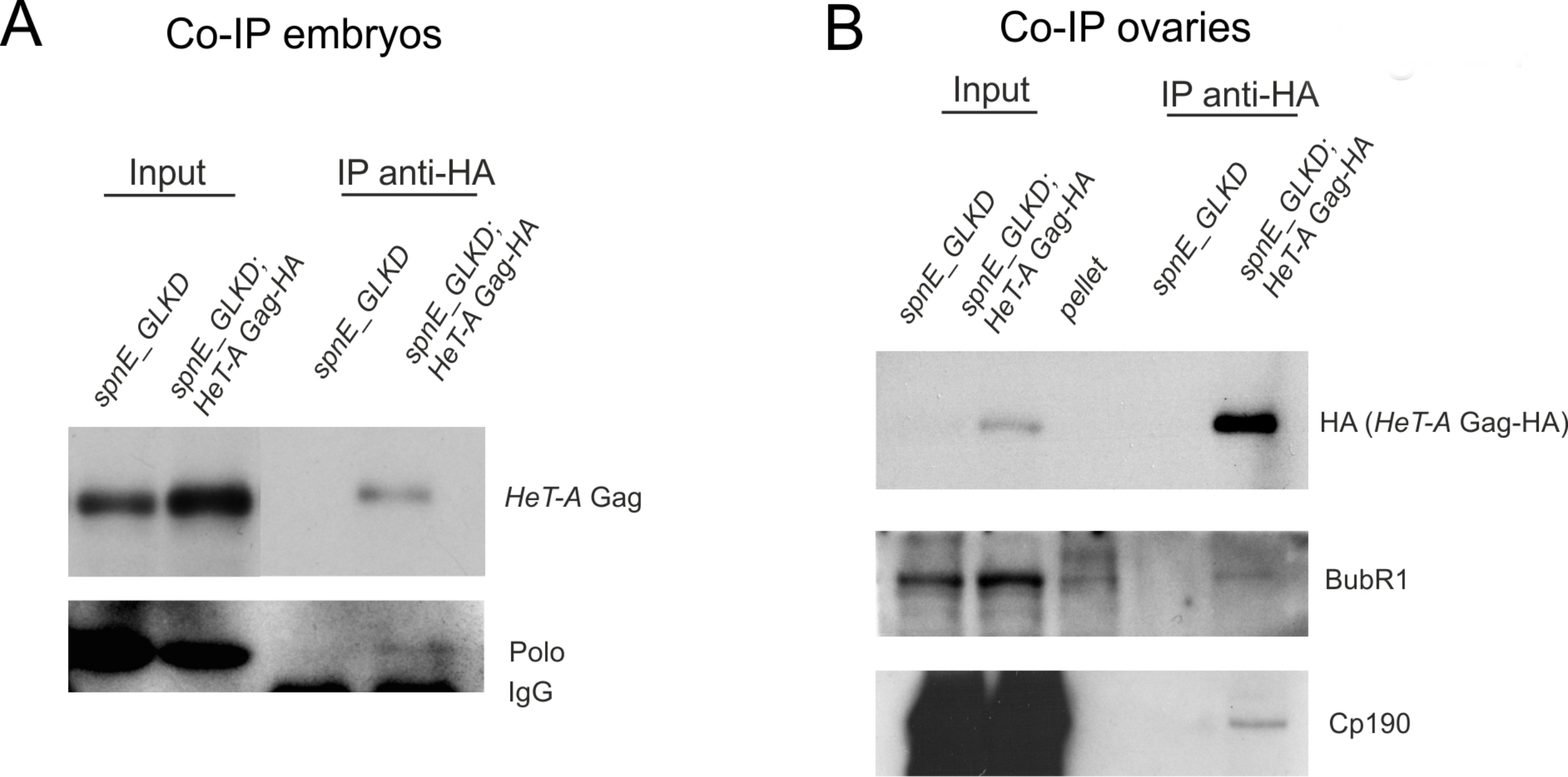

Supplement: S4 Fig — (A) Co-IP experiment performed on extracts from 0-2-h-old spnE_GLKD embryos expressing HeT-A Gag-HA reveals that Polo is co-purified with HeT-A Gag-HA. (B) Co-IP experiment performed on extracts from spnE_GLKD ovaries expressing HeT-A Gag-HA reveals that BubR1and CP190 are co-purified with HeT-A Gag-HA. Post-extraction insoluble pellet was loaded between Input and IP probes. The antibodies used for co-IP and western blotting are indicated on the right. (TIF) [file pone.0258156.s007.tif]

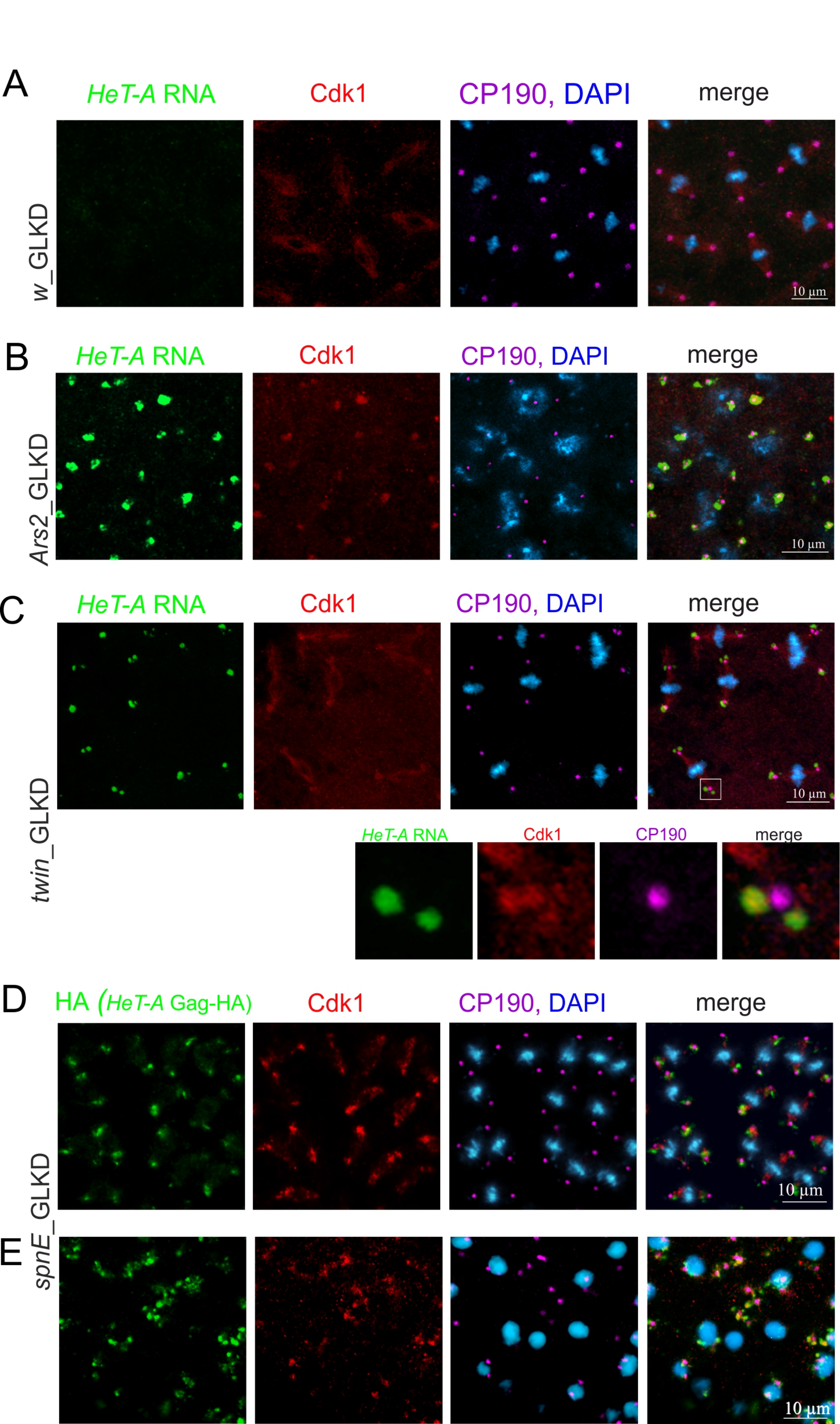

Supplement: S5 Fig — HeT-A RNA FISH (green) and coimmunostaining of Cdk1 (red) and CP190 (magenta) in the control (A), Ars2_GLKD (B) and twin_GLKD (C) early syncytial embryos. Lower panels in (C) show the enlarged area around the centrosome in twin_GLKD embryo. (D, E) Coimmunostaining of HeT-A Gag-HA (green), Cdk1 (red) and CP190 (magenta) in the spnE_GLKD is shown. Free centrosomes in the embryo cortex are shown (E). Blue, DNA. (TIF) [file pone.0258156.s008.tif]

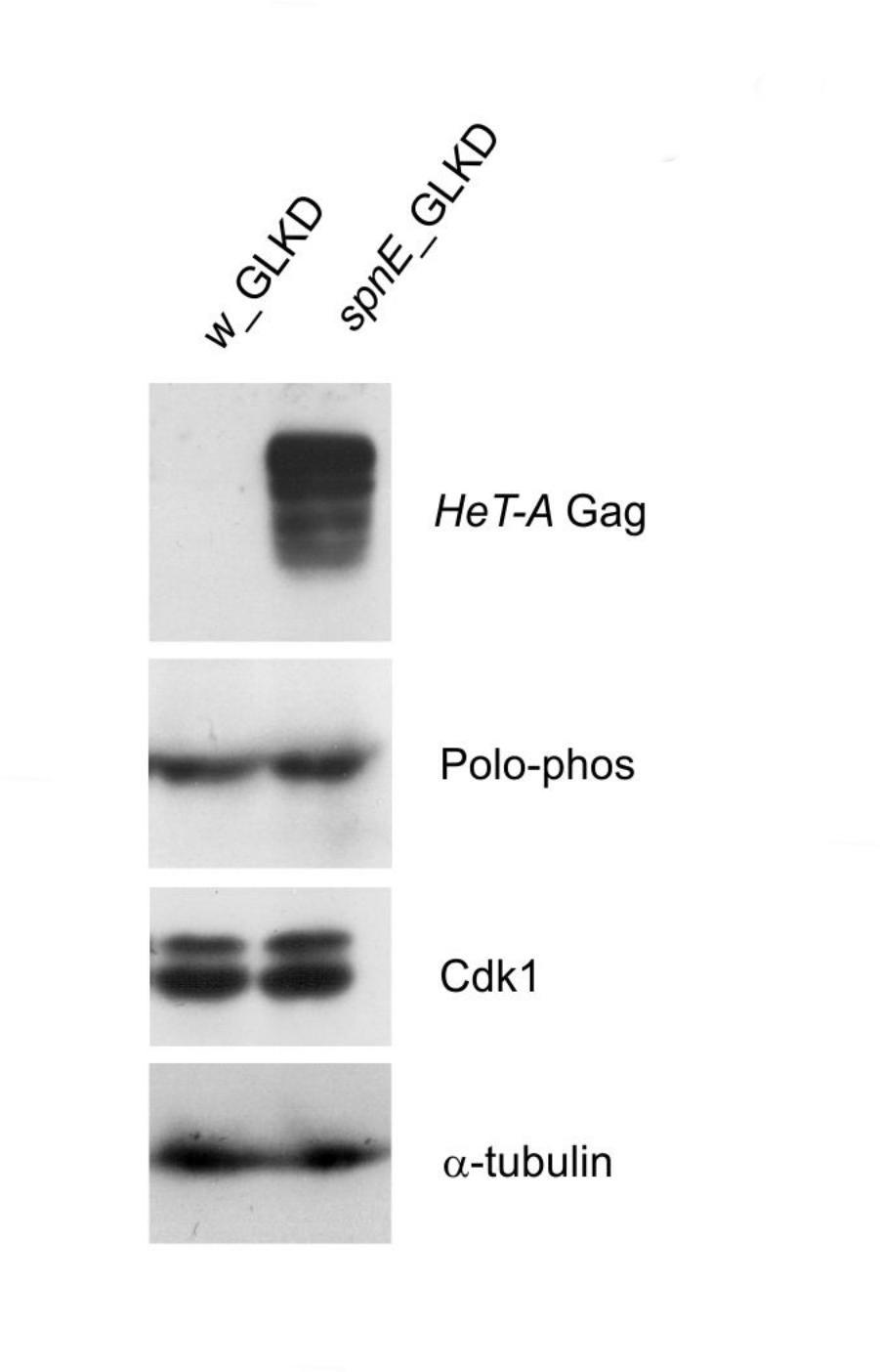

Supplement: S6 Fig — Western blotting of 0–2 h old embryo lysates shows the overexpression of HeT-A Gag and unchanged Polo and Cdk1 levels in spnE_GLKD embryos compared with the control (w_GLKD). Antibodies are indicated on the right. Anti-Plk1 antibodies (pThr210, LSBio) used here recognize phosphorylated form of Polo. (TIF) [file pone.0258156.s009.tif]

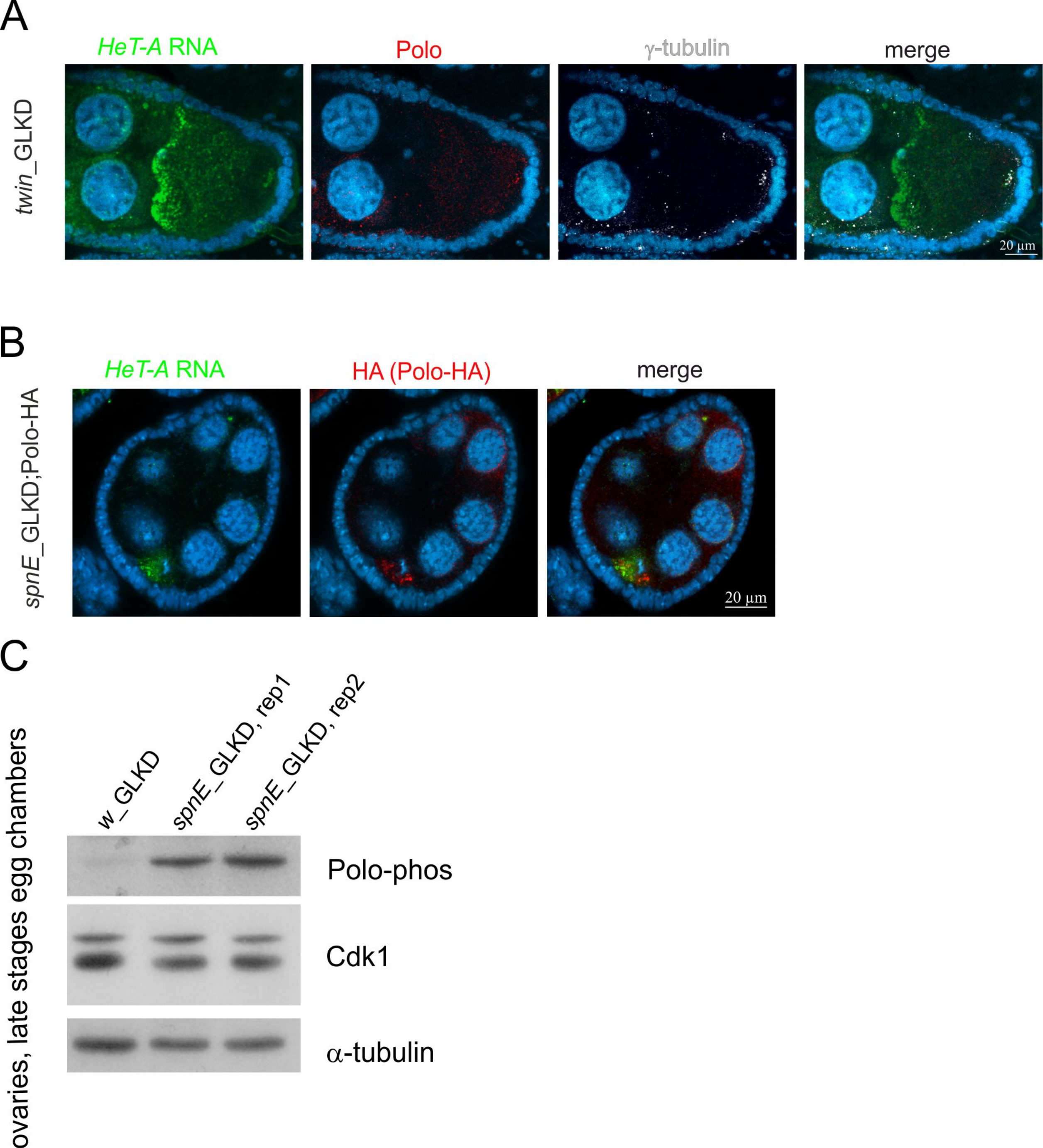

Supplement: S7 Fig — (A) Colocalization of HeT-A RNA (green), Polo (phosphorylated form, red) and γ-tubulin (grey) in the oocyte of twin_GLKD at stage 9 is shown. Blue, DNA. (B) Accumulation of Polo-HA (red) and HeT-A Gag (green) in the oocyte of spnE_GLKD expressing Polo-HA at stage 6 of oogenesis is shown. Blue, DNA. (C) Western blotting confirms accumulation of phosphorylated Polo in spnE_GLKD egg chambers at 12–14 stages (rep1 and rep2, two biological replicates) compared with w_GLKD. The antibodies used are indicated on the right. (TIF) [file pone.0258156.s010.tif]

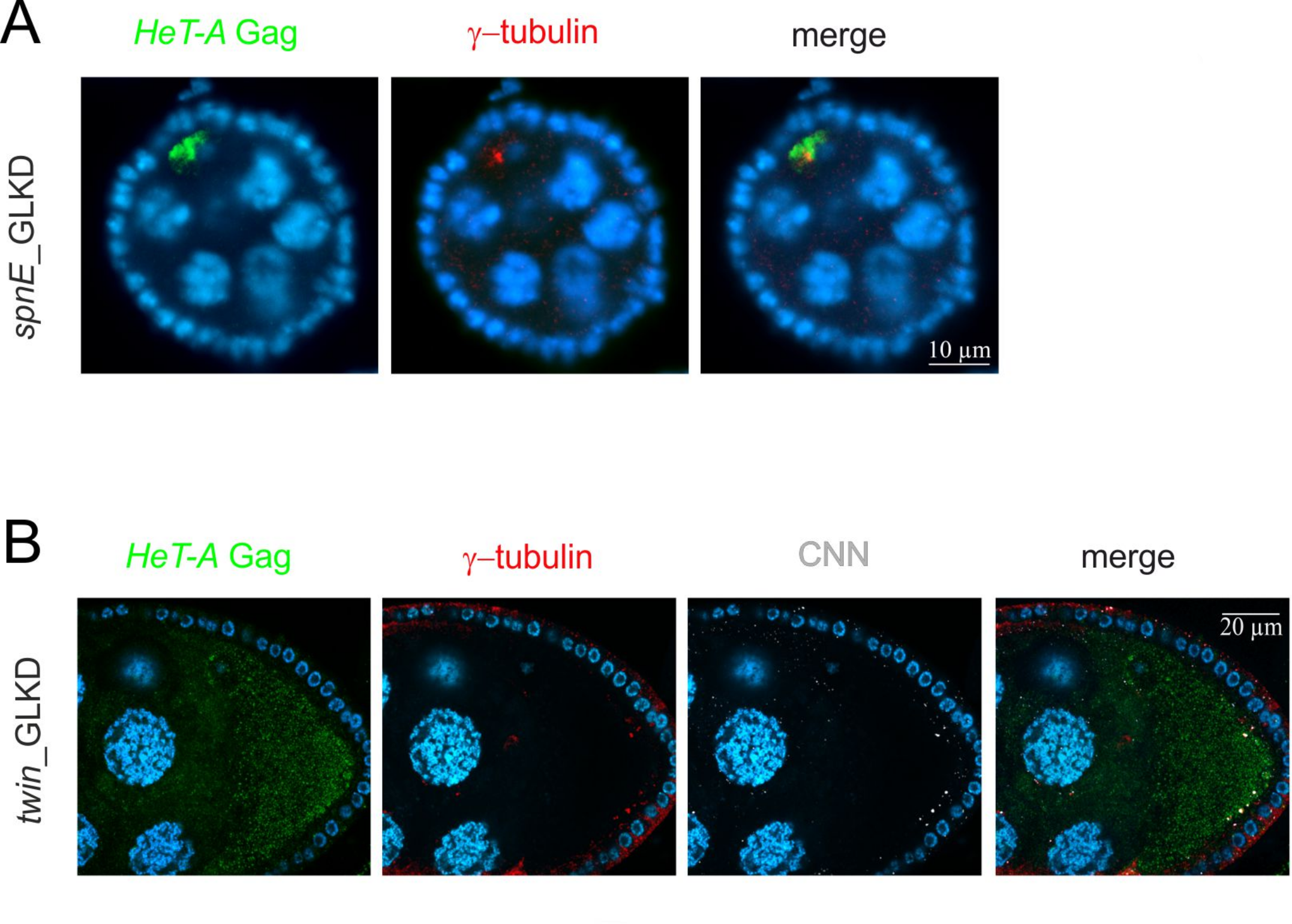

Supplement: S8 Fig — (A) Coimmunostaining demonstrating colocalization of HeT-A Gag (green) and γ-tubulin (red) in spnE_GLKD ovaries at stage 5 of oogenesis is shown. (B) Immunostaining reveals accumulation of HeT-A Gag (green) and multiple γ-tubulin (red) and CNN (grey) foci at stage 9 of oogenesis in twin_GLKD. Blue, DNA. (TIF) [file pone.0258156.s011.tif]

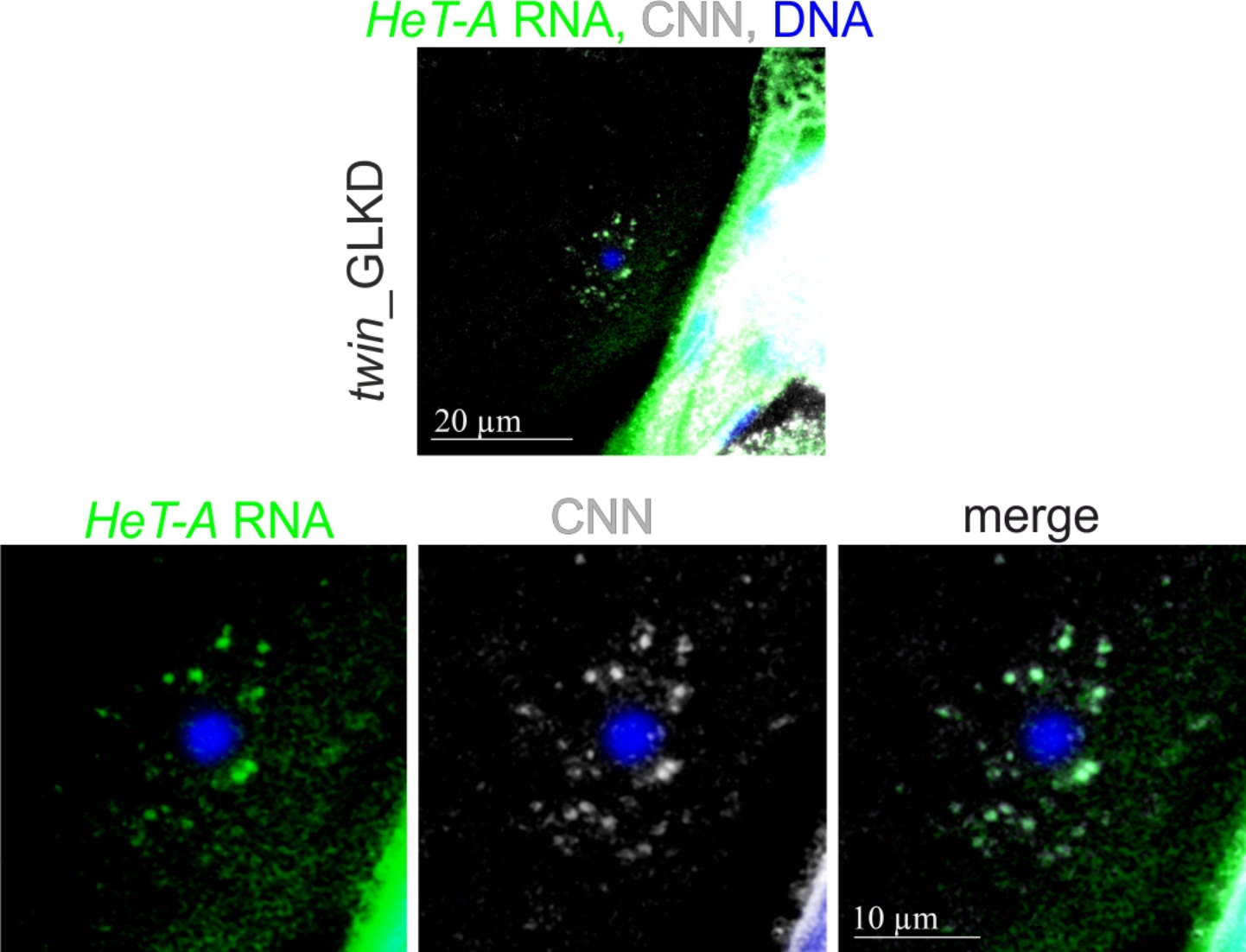

Supplement: S9 Fig — HeT-A RNA FISH (green) combined with CNN (gray) immunostaining in twin_GLKD at stage 13–14 egg chamber. The lower panel is a blow up of the area around the oocyte nucleus (the upper panel). DNA, blue. (TIF) [file pone.0258156.s012.tif]
